# Phenotyping: Using Machine Learning for Improved Pairwise Genotype Classification Based on Root Traits

**DOI:** 10.3389/fpls.2016.01864

**Published:** 2016-12-06

**Authors:** Jiangsan Zhao, Gernot Bodner, Boris Rewald

**Affiliations:** ^1^Department of Forest and Soil Sciences, University of Natural Resources and Life SciencesVienna, Austria; ^2^Division of Agronomy, Department of Crop Sciences, University of Natural Resources and Life SciencesVienna, Austria

**Keywords:** breeding, cultivar classification, pea (*Pisum sativum* L.), random forest (RF), root phenotyping, root trait selection, support vector machine (SVM)

## Abstract

Phenotyping local crop cultivars is becoming more and more important, as they are an important genetic source for breeding – especially in regard to inherent root system architectures. Machine learning algorithms are promising tools to assist in the analysis of complex data sets; novel approaches are need to apply them on root phenotyping data of mature plants. A greenhouse experiment was conducted in large, sand-filled columns to differentiate 16 European *Pisum sativum* cultivars based on 36 manually derived root traits. Through combining random forest and support vector machine models, machine learning algorithms were successfully used for unbiased identification of most distinguishing root traits and subsequent pairwise cultivar differentiation. Up to 86% of pea cultivar pairs could be distinguished based on top five important root traits (Timp5) – Timp5 differed widely between cultivar pairs. Selecting top important root traits (Timp) provided a significant improved classification compared to using all available traits or randomly selected trait sets. The most frequent Timp of mature pea cultivars was total surface area of lateral roots originating from tap root segments at 0–5 cm depth. The high classification rate implies that culturing did not lead to a major loss of variability in root system architecture in the studied pea cultivars. Our results illustrate the potential of machine learning approaches for unbiased (root) trait selection and cultivar classification based on rather small, complex phenotypic data sets derived from pot experiments. Powerful statistical approaches are essential to make use of the increasing amount of (root) phenotyping information, integrating the complex trait sets describing crop cultivars.

## Introduction

A policy report of the European Union noted recently that protein crops, e.g., bean, lentil, lupine, pea, and soya, are currently grown on 1.8% of arable land in the EU only, compared with 4.7% in 1961, and about 8% in Australia and Canada, 14.5% in North America, and 25.5% in South America (Jiang et al., 2004; EU, 2013; FAOSTAT, 2014; Cernay et al., 2015). This is despite grain legumes representing a significant source of protein for food (Vaz Patto et al., 2014; Multari et al., 2015) and feed (Jezierny et al., 2010; Koivunen et al., 2014), and legume cultivation reducing the need for N fertilizer even for subsequent crops in the rotation (Preissel et al., 2015). Recent studies have identified a comparative lack of breeding investment in Europe to improve grain legume adaptation to local agro-climatic conditions and management techniques (Annicchiarico and Iannucci, 2008; Lizarazo et al., 2015). While distinct leguminous crops are used locally for food and feed, and local cultivars are kept in numerous collections in gene banks, research institutions, and also in farms/home gardens, this genetic pool cannot be used at its full potential for large-scale agriculture and breeding programs until important traits have been determined.

Plant phenotyping intends measuring complex traits related to growth, yield, and adaptation to stress at different macroscopically scales of plant organization (Fiorani and Schurr, 2013). Examples for measured parameters are leaf vein structure (Sack and Scoffoni, 2013; Caringella et al., 2015), photosynthetic efficiency (Gilbert et al., 2011; Gorbe and Calatayud, 2012; Grady et al., 2013), root morphology (Iyer-Pascuzzi et al., 2010; Bucksch et al., 2014), biomass (Stachowicz et al., 2013; Poorter et al., 2015), and yield quantity and quality (Iqbal and Lewandowski, 2014).

Especially the modification of root system architecture (RSA) could contribute to improvements of desirable agronomic traits such as yield, drought tolerance, and resistance to nutrient deficiencies; thus, RSA was described key to a second green revolution improving resource use efficiency of crops (Lynch, 2007). For example, an architectural trait enhancing topsoil foraging is a higher number of basal roots – contributing significantly to phosphorous acquisition (Lynch, 2011), while deep rooting can, e.g., sustain water acquisition during drought periods or improve uptake of percolating nitrate (Pinheiro et al., 2005). Recently, an increased focus was laid on improving high-throughput, image-based root phenotyping approaches (Berger et al., 2010; Hartmann et al., 2011; Mairhofer et al., 2012; Fiorani and Schurr, 2013; Li et al., 2014). See Kuijken et al. (2015) for a recent review on the latest developments in root phenotyping and an overview on environmental and genetic factors influencing root phenotypes.

Advanced machine learning (ML) approaches encompass promising statistical tools for variable selection and group classification. While the use of ML approaches in non-genetic/biochemical plant sciences is still scarce, ML was introduced to promote RSA classification in the recent past (Zhong et al., 2009; Iyer-Pascuzzi et al., 2010). Iyer-Pascuzzi et al. (2010) use of ML methods mainly benefited from using a non-invasive imaging system which enabled them to acquire 16 traits from a high number of pictures [∼200 (pseudo-)replicates per genotype]. However, the limited plant age and highly artificial growth conditions are major disadvantages of many non-invasive and high-throughput root phenotyping methods (Bucksch et al., 2014): RSA differs with ontogeny (Hargreaves et al., 2009; Wojciechowski et al., 2009; Hund et al., 2011) and is highly plastic to edaphic conditions (Tracy et al., 2012; Rich and Watt, 2013). Thus, analyses on mature plants *in situ* or under more realistic growth conditions, which mostly rely on manual, destructive methods [e.g., ‘shovelomics’; Trachsel et al. (2011)] continue to be essential although the number of replicates and measurements is often limited but the complexity of variables (i.e., traits) remains high. Similar to the situation in cell biology (Sommer and Gerlich, 2013), available ML approaches in plant sciences have been optimized for large-scale screenings, probably partially due to the difficulty in applying ML algorithms with unbiased variable selection on low number of replicates (Bucksch et al., 2014).

Among supervised ML algorithms, random forest (RF) is a non-parametric method with high accuracy and robustness to noise (Breiman, 2001). RF has been applied in several biological fields, like gene (Díaz-Uriarte and De Andres, 2006) and protein sequence (Pan and Shen, 2009) selection, and disease prediction (Yang et al., 2014). However, most previous studies mainly focused on improving classification accuracy with variable selection rather than variable interpretation (Liu et al., 2014; da Costa et al., 2015; Gowin et al., 2015) because the variable importance measure is biased in the standard RF algorithm – overestimating the importance of correlated predictor variables (Strobl and Zeileis, 2008). However, unbiased variable selection is essential to stable classification and meaningful interpretation of plant traits and other data and can be achieved by using an improved RF algorithm – based on a conditional permutation scheme as a computational means to determine variable importance (Strobl and Zeileis, 2008; Strobl et al., 2008). Support vector machines (SVMs) are another set of supervised ML methods which can be trained to classify individuals in high-dimension space (Cortes and Vapnik, 1995). SVMs have been widely used in neuro-image classification (Gaonkar and Davatzikos, 2013) and face detection (Shan, 2012). SVMs can be differentiated based on kernel functions (Okkan and Serbes, 2012): linear kernel functions (linear SVMs) were previously used for variable selection of root systems (Iyer-Pascuzzi et al., 2010). However, variable selection by ranking absolute values of weights are biased, as the absolute weight values of irrelevant variables can be as high as of important ones (Statnikov et al., 2006; Gaonkar and Davatzikos, 2013). SVMs based on Gaussian radial basis function (rbf) kernel often provide a better performance on ‘noisy’ data sets not separable linearly – resulting in the more widely use of rbf SVMs in classification (Hsu et al., 2010).

Powerful statistical approaches are essential to make use of the increasing amount of (root) phenotyping information, integrating the complex trait sets (describing RSA). Combining RF with rbf SVMs for variable selection and group classification, respectively, might overcome problems in applying ML approaches on data sets characterized by a rather low signal-to-noise ratio such as manually derived phenotyping data (Liu et al., 2004). For example, Löw et al. (2012) found a significantly higher classification accuracy of pre-crops in two out of four agricultural regions using satellite images when applying a combination of RF and SVMs compared to using either RF or SVMs for both trait selection and classification. Thus, aims of this study were to determine if (i) RF can be reproducible used for selecting important root traits (i.e., root traits distinguishing mature pea cultivars), and (ii) how root trait selection influences cultivar classification by rbf SVMs. We hypothesize that rbf SVMs classification is superior to traditional univariate tests if important root traits are identified by RF. *Pisum sativum* L. was selected as test species because it is one of the most frequently cultivated grain legumes worldwide (Alves-Carvalho et al., 2015), with especially European genetic resources still insufficiently characterized.

## Materials and Methods

### Plant Material and Experimental Set-Up

Sixteen randomly selected cultivars of pea (*P. sativum* L.) were used for root phenotyping (**Table 1**), originating either from Southern (Portugal and Spain) or Northern Europe (Estonia, Latvia, Norway, and Sweden). Seeds were provided by partners within the EU FP7 project ‘Eurolegume’ and by the Nordic gene bank.

**Table 1:** Sixteen pea (*Pisum sativum* L.) cultivars used locally for food in different European countries and institutions donating the seeds for the experiment.

Abbreviation	Cultivar	Country of origin	Donor institution
ps1.Estonia	Eesti hall	Estonia	ECRI
ps2.Estonia	Eesti kollane soogihernes	Estonia	ECRI
ps3.Estonia	Jõgeva roheline	Estonia	ECRI
ps4.Estonia	Seko	Estonia	ECRI
ps5.Latvia	Alma	Latvia	SPPBI
ps6.Latvia	Bruno	Latvia	SPPBI
ps7.Latvia	k-4833 Stendes Hero	Latvia	SPPBI
ps8.Latvia	Retrija	Latvia	SPPBI
ps9.Norway	NGB 10778.1	Norway	NordGen
ps10.Norway	NGB 20045.3	Norway	NordGen
ps11.Portugal	Gp 3263	Portugal	INIAV
ps12.Portugal	Gp 3491	Portugal	INIAV
ps13.Portugal	Gp 3497	Portugal	INIAV
ps14.Portugal	Grisel	Portugal	INIAV
ps15.Sweden	NGB 1025131	Sweden	NordGen/ Uppsala U
ps16.Sweden	NGB 131381	Sweden	NordGen/ Uppsala U


*ECRI, Estonia Crop Research Institute; NordGen, Nordic Genetic Resource Center, Norway; INIAV, Instituto Nacional de Investigação Agrária e Veterinária, Portugal; SPPBI, Priekuli Plant Breeding Institute, Latvia; Uppsala U, Uppsala University, Sweden.*

Experiments were conducted in a large plastic foil greenhouse from June 13th, 2014 to October 7th, 2014 located in Tulln, Austria (48.33°N, 16.05°E). Aeration openings of the greenhouse were fitted with mesh to prevent insect infestations. Solar radiation, air temperature, and relative humidity were hourly recorded 2 m above ground; mean air temperature during the measurement period was 20.7°C, relative humidity ranged between 18.6–87.7% with a mean of 59.9%. Mean daily sum of solar radiation was 13.28 MJ m^-2^ day^-1^, with a maximum of 27.41 MJ m^-2^ day^-1^ at July 1st; day length (solar radiation ≥120 W m^-2^) varied between 10 and 16 h.

Seeds of all cultivars were germinated in a growth chamber (Fitotron; Weiss-Gallenkamp, UK) at 25 ± 1°C. Seeds were coated with a rhizobium suspension (Steinberga et al., 2008) before being planted in 0.5-L plastic bags (10 cm high) filled with washed quartz sand (0.7–1.2 mm-sized) amended with 1 g of slow release fertilizer (Osmocote Pro 3-4M; 17-11-10+2MgO+TE; ICL Specialty Fertilizers, Tel Aviv, Israel). Initial germination was conducted in darkness; after the first seed germinated, light (PAR 350 μE m^-2^ s^-1^) was turned on (16 h light/8 h dark). Germination time varied between 4 and 6 days with minor differences between cultivars (data not shown). After 10–14 days, eight similar-sized seedlings per cultivar were selected for transplanting.

In the greenhouse, eight blocks of 16 plastic tubes each (128 tubes in total) were established on wooden frames in North to South direction. The plastic tubes used as pots/growing cylinders in the experiments were 108 cm long and 20 cm in diameter (∼32 L); the bottom was sealed with a cap; holes covered with a glass fiber mat allowed for free drainage. Before the tubes were filled with washed, 0.7–1.2 mm-sized quartz sand, a plastic liner was installed in each tube allowing for undisturbed removal of the substrate during harvest; the liner was perforated at the bottom 10 cm. Measurements in large plastic tubes have previously shown good agreement with maximum rooting depth and root length density as determined in the field and have been used to explore root traits in other legumes such as chickpea (Kashiwagi et al., 2006; Vadez et al., 2008). For transplanting, germination bags were placed inside the tubes and cut open at the side and bottom to prevent root disturbance. One plant per cultivar was randomly arranged in each of the eight blocks. 8.3 g of an AMF inoculum (*Glomus mosseae* BEG95, *G. intraradices*, and *G. geosporum* BEG199; supplied by Dr. Aleš Látr, Symbiom, Czech Republic) were added to each plant individual around the root systems at depths of 0–10 cm before the tube was brimmed with additional sand. An automated, pressure-compensated drip-irrigation system was used to supply all plants with ample amounts of water and a modified Long Ashton nutrient solution (Jia et al., 2004); amounts were adjusted to increasing plant size and weather conditions.

### Harvest and Analysis

Plants were randomly harvested within blocks at 71–92 days after transplanting. After harvesting the shoots (data not shown), the tubes were placed horizontally and the plastic liner was pulled out on a 1.5 mm-mesh table. After the plastic liner was cut open, roots were then manually excavated as previously described by Kashiwagi et al. (2005) and others. No roots reached the bottom of the tube and few roots were discovered at the sides, indicating a rather unrestricting pot size. After the root system was uncovered, the maximum rooting depth was determined. It was further washed and rinsed in a bucket filled with clean tap water (Miguel, 2012), photographed next to a size standard, stored in a water-filled plastic bag, and transported to the lab for further analysis. Detached (i.e., shed/broken off) root segments were accurately collected from the remaining sand on the mesh table (mesh size: 2 × 2 mm), stored in paper bags and transported to the lab, oven dried (65°C, 48 h) and added to root biomass. In the lab, the root systems were stored at 4°C until further analysis (≤3 weeks) took place (Hu et al., 2013).

For in-depth architectural and morphological analysis, the root systems of 5–7 plant individuals per cultivar (97 plants in total) were manually dissected into tap root and laterals. Laterals along the tap root and the tap root were separated into the five depth classes 0–5, 5–10, 10–20, 20–40, and 40-100 cm. Three lateral root samples from the depth classes 0–5, 5–10, and 10–20 cm along the tap root were scanned in water-filled trays (Epson Expression 10000XL; Epson, Nagano, Japan) at 400 dpi, grayscale. Pictures were analyzed for diameter, surface area, length, and volume with the PC program WinRhizo 2012b Pro (Régent Inst., Ville de Québec, QC, Canada). Subsequently, all root samples were dried (65°C, 48 h) and weighed to an accuracy of ± 0.1 mg (CP225D; Sartorius, Göttingen, Germany). The specific root area (SRA, cm^2^ g^-1^), specific root length (SRL, cm g^-1^), tissue density (TD, g cm^-3^), the total root surface area (totalRSA), root length (RL), and root volume were calculated. Determined root traits (Guo et al., 2008; Rewald et al., 2011) are listed in **Table 2** (Cramer et al., 2007; Alves-Carvalho et al., 2015).

**Table 2:** Abbreviation and description of 36 root traits derived either from direct measurement or from calculation after manual phenotyping of 16 European *P. sativum* cultivars.

Abbreviation	Unit	Definition	Level of trait
slatdiam2.5	mm	The average diameter of single lateral root originated from 0 to 5 cm deep tap root	Lateral roots
slatlth2.5	cm	The average length of single lateral root originated from 0 to 5 cm deep tap root	
slatsa2.5	cm^2^	The average surface area of single lateral root originated from 0 to 5 cm deep tap root	
sLSRL2.5	cm g^-1^	The average specific root length (root length divided by dry biomass) of single lateral root originated from 0 to 5 cm deep tap root	
sLSRA2.5	cm^2^ g^-1^	The average specific root area (root area divided by dry biomass) of single lateral root originated from 0 to 5 cm deep tap root	
sLTD2.5	g cm^-3^	The average tissue density (root dry biomass divided by root volume) of single lateral root originated from 0-5 cm deep tap root	
slatdw2.5	g	The average dry weight of single lateral root originated from 0 to 5 cm deep tap root	
latRL2.5	cm	The length of all the lateral roots originated from 0 to 5 cm deep tap root	
latRL7.5	cm	The length of all the lateral roots originated from 5 to 10 cm deep tap root	
latRSA2.5	cm^2^	The surface area of all the lateral roots originated from 0 to 5 cm deep tap root	
latRSA7.5	cm^2^	The surface area of all the lateral roots originated from 5 to 10 cm deep tap root	
latRV2.5	cm^3^	The volume of all lateral roots coming originated from 0 to 5 cm deep tap root	
latRV7.5	cm^3^	The volume of all lateral roots coming originated from 5 to 10 cm deep tap root	
latRDW2.5	g	All lateral roots dry weight from 0 to 5 cm deep tap root	
latRDW7.5	g	All lateral roots dry weight from 5 to 10 cm deep tap root	
latRDW	g	The dry weight of all lateral roots	
latn2.5		The number of lateral roots originated from 0 to 2.5 cm deep tap root	
latn5		The number of lateral roots originated from 2.5 to 5 cm deep tap root	
latn7.5		The number of lateral roots originated from 5 to 10 cm deep tap root	
tapdiam2.5	mm	The diameter of tap root from 0 to 5 cm depth	Tap root
tapdiam7.5	mm	The diameter of tap root from 5 to 10 cm depth	
tapTD2.5	g cm^-3^	The tissue density of tap root from 0 to 5 cm depth	
tapTD7.5	g cm^-3^	The tissue density of tap root from 5 to 10 cm depth	
tapdw2.5	g	Tap root dry weight from 0 to 5 cm depth	
tapdw7.5	g	Tap root dry weight from 5 to 10 cm depth	
tapRL	cm	Tap root length	
tapRSA	cm^2^	Tap root surface area	
tapRV	cm^3^	Tap root volume	
tapRDW	g	Tap root dry weight	
lateraltapRDWR	g g^-1^	The ratio of all lateral roots dry weight and tap root dry weight	Root system
lateraltapSAR	cm^2^ cm^-2^	The ratio of all lateral roots area to tap root area	
totalRSA	cm^2^	Total root surface area of whole root system	
totalRL	cm	Total root length of whole root system	
totalRV	cm^3^	Total root volume of whole root system	
rootdw	g	Root dry weight	
rootdep	cm	Root depth	


### Data Analysis

Thirty-six root traits in total, either directly measured or calculated, were available for analysis (**Table 2**). Non-normal distributed root traits were box-cox transformed with ‘MASS’ package, version 7.3-44, in R for Windows version 3.2.2 (R Core Team, 2015). Multiple imputation was conducted by ‘Amelia’ package, version 1.7.3. The R code used for data preparation can be found as **Supplementary Method S1**.

#### Random Forest and Support Vector Machines

Random forest (Breiman, 2001; Strobl et al., 2009) was used to rank root traits according to their importance for classification; SVMs (Vapnik and Vapnik, 1998) were used for multiclass or pairwise cultivar classification. A flow chart outlining data handling steps can be found as **Supplementary Method S2**.

Because the multiclass classification resulted in very low accuracy (see **Supplementary Figure S1**), only the pairwise classification was pursued further. In order to use the ‘cforest’ function in the R ‘Party’ package (version 1.0–23) for root traits importance measure, individuals of each pair of cultivars were oversampled four-times (to gain the number of data points required by the software algorithm) for pairwise classification. As RF was used for traits important measure only, the whole oversampled data set was used. Afterward, the number of root traits randomly chosen at each split (building each tree), *mtry*, was tuned. Even though it has been suggested that mtry = $\sqrt{n}$, *n* is the amount of root traits, always generates acceptable classification accuracy (Díaz-Uriarte and De Andres, 2006), the accuracy might vary (Verikas et al., 2011). Thus, 1000 trees with 14 *mtry*s (i.e., using 1, 2, 4, 6, 8, 10, 12, 14, 16, 18, 20, 24, 28, or 32 traits), were constructed in RF to determine the importance of each root trait in each pairwise comparison. In a third step, root traits were ranked in each pair based on their importance. Root traits importance was calculated in RF based on unbiased conditional inference permutation test (Strobl and Zeileis, 2008; Ball et al., 2014). The most important root trait was defined as the one leading to the highest mean decrease of classification accuracy when values of a variable are randomly permuted across all 1000 trees (Breiman, 2001). Because negative importance values are due to random variation around zero (Strobl et al., 2009), values of root traits importance were first subtracted by the absolute value of the lowest negative importance and then normalized between 0 and 1 before being ranked in each pair.

Cultivar classification by either SVMs or RF were conducted through passing different combinations of top ranking important root traits derived from RF to SVMs/RF classification models. In order to find the combination of top ranking important root traits (*Timp*) that generate SVMs/RF models with the highest overall prediction accuracy, different numbers of *Timp*s (Timp*i*) were tested in SVMs/RF classification. Even though the number of variables in final SVMs models should generally be <10 (Nicodemus et al., 2010), nine trait combinations were tested: top *i* important root traits (*Timpi* = 2, 3, 5, 7, 9, 11, 13, 15) and all root traits (36). In rbf SVMs models, twelve kernel parameter *C* and regularization parameter *gamma* (Meyer, 2015) from 10^-5^ to 10^6^ were tuned; the best combination of *C* and *gamma* leading to the highest prediction accuracy was chosen through leave-one-out cross-validation (LOOCV; Kohavi, 1995). Each SVMs pairwise classification model was validated with LOOCV. The accuracies of RF classification were derived from out of bag error (OOB); *Timp* = 1 was used to find the maximum HACCs when tuning different *mtrys* and *Timps* combinations. The validation accuracy of models means whether the different labeled observations were accurately classified.

$$\text{Accuracy = }\frac{\text{TP+TN}}{\text{TP+TN+FP+FN}}$$

**FIGURE 1:**
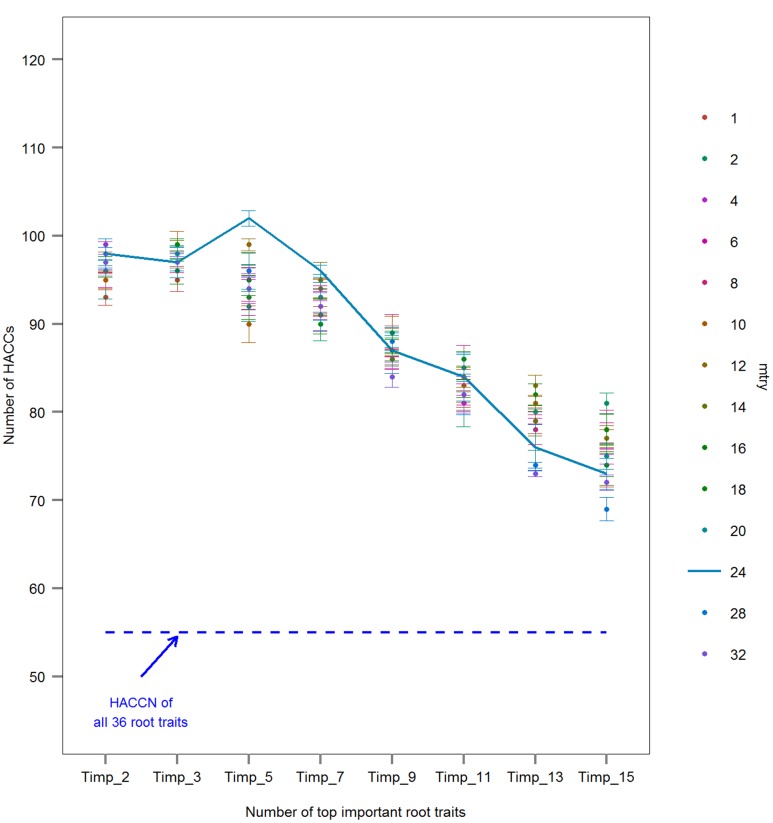
**Comparison of the number of high accuracy classifications (HACCs; prediction accuracy ≥80%) using rbf SVMs models with different numbers of *Pisum sativum* root traits randomly chosen for each tree, *mtry*s, and different number of top important root traits, *Timp_i* = 2, 3, 5, 7, 9, 11, 13, 15 (mean ± SE; *n* = 3 runs).** One hundred twenty pairs of 16 European *P. sativum* cultivars were analyzed. A dashed blue line represents the number of HACCs (HACCN) retrieved using all 36 root traits in SVMs classification. A line connecting HACC numbers derived by using different number of *Timp*s at constant *mtry* = 24 was added for visualization.

Where TP is the number of true positives, TN is the number of true negatives, FP is the number of false positives, and FN is the number of false negatives. The validation accuracy was treated as final prediction accuracy of SVMs/RF classifications. Classifications with an average prediction accuracy ≥80% were regarded as a high accuracy classifications (HACCs); the 80% level was determined acceptable by previous ML studies (Wang et al., 2010; Liu et al., 2014; Shang and Chisholm, 2014; Zheng et al., 2014; Sacchet et al., 2015). The whole process – RF ranking of root traits in each cultivar pair, SVMs and RF classification of pairs using different *mtrys* and *Timp*s – was repeated three times; the average accuracy with standard error was calculated. The combination of different *mtry*s and different *Timpi*s generating the highest average accuracy of SVM/RF models was treated as optimal *mtry* and *Timpi* combination; the frequencies of the corresponding top five important root traits (T5IRT) from all HACCs were calculated. Because SVM models yielded higher classification accuracies than RF (**Figure 1**; **Supplementary Figure S2**), classification by RF was not pursued further. Subsequently the accuracies of SVMs models derived from Timp5 were compared to six runs of randomly selected subsets of five root traits each (R_5.1–R_5.6) to determine the benefits of root trait selection based on RF for cultivar classification.

The R for Windows package ‘party’ (v. 1.0–23) was used for RF trait ranking; the R package ‘e1071’ (v. 1.6–7) was used for rbf SVMs classification (data scaled), the ‘randomForest’ package (v. 4.6–12) for RF classification. The R code used for RF root traits ranking and rbf SVMs classification can be found as **Supplementary Method S3**.

#### Univariate Permutation Test

In order to compare the efficiency between a univariate test and the combination of RF and rbf SVMs in cultivar classification, an exact permutation test (Hesterberg et al., 2005) was carried out (α = 0.05), with both Bonferroni and fdr correction (Benjamini and Hochberg, 1995), to compare the root traits involved in each pairwise classification. The R code used can be found as **Supplementary Method S4**.

## Results

### RF Root Traits Selection and SVMs Classification

**FIGURE 2:**
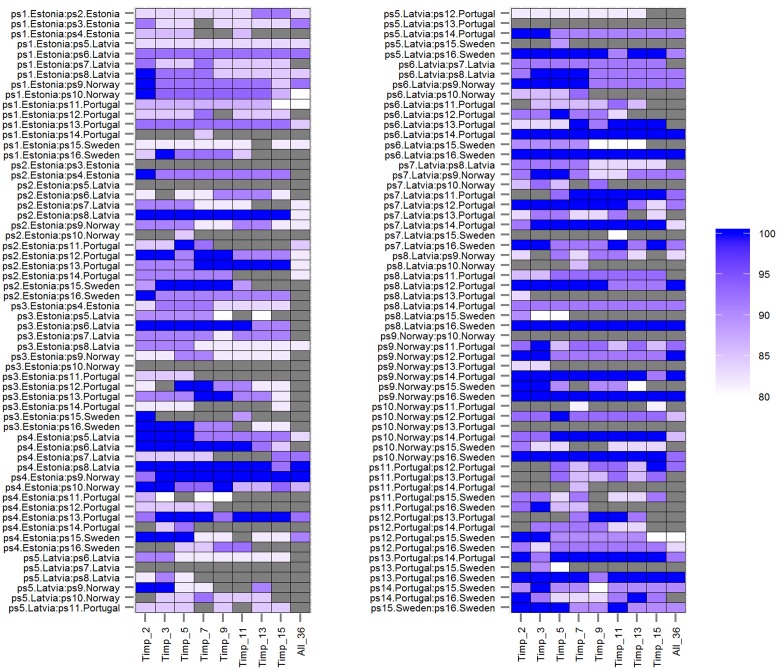
**Prediction accuracies of rbf SVMs models (*mtry* = 24) using different numbers of top important root traits (*Timp_i* = 2, 3, 5, 7, 9, 11, 13, 15) and all (36) root traits (*All_36*) for 120 pairwise comparisons among 16 European *P. sativum* cultivars.** Prediction accuracies from 80% (white) to 100% (blue) are shown as color gradient; lower accuracies are displayed in gray. Cultivars are listed in **Table 1**.

**FIGURE 3:**
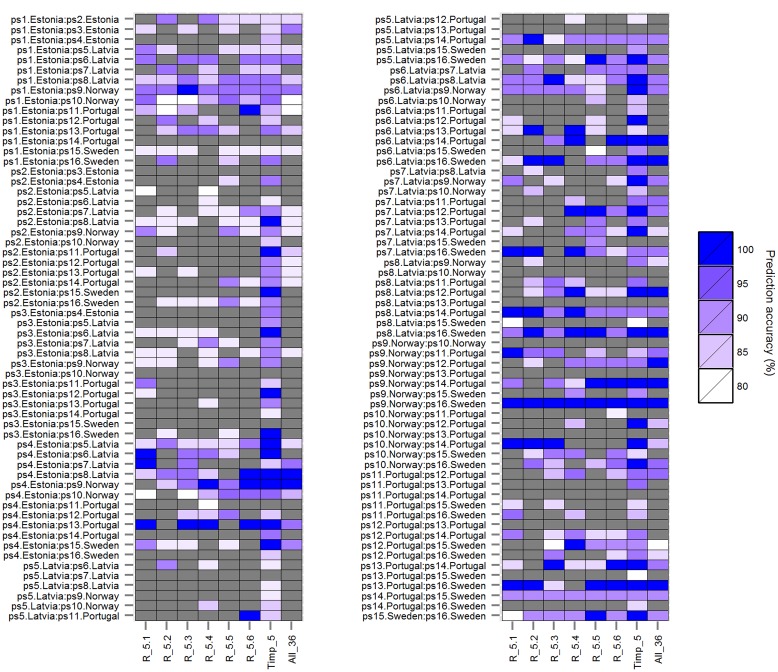
**Prediction accuracies of rbf SVMs models (*mtry* = 24) using five randomly selected root traits each in six runs (*R_5.1, R_5.2, R_5.3, R_5.4, R_5.5*, and *R_5.6*) compared to prediction accuracies using top five important root traits (*Timp_5*), and all (36) root traits (All_36) for 120 pairwise comparisons among 16 European *P. sativum* cultivars.** Prediction accuracies from 80% (white) to 100% (blue) are shown as color gradient; lower accuracies are displayed in gray. Cultivars are listed in **Table 1**.

The classification accuracy of SVMs multiclass classifications with and without root trait selection were 16.5 and 22.7%, respectively (**Supplementary Figure S1**), thus a pairwise approach was pursued thereafter. Similarly, the number of HACCs generated from different *mtry*s and *Timpi*s combinations in pairwise RF (77 HACCs with *mtry* = 32, *Timpi*s = 2; **Supplementary Figure S2**) were much lower compared to the classification accuracy achieved by pairwise SVMs (see below and **Figure 1**). Thus, this RF/RF approach was not followed further but a combination of RF (for trait ranking) and SVMs (for classification) was used. Through testing a series of combinations of different *mtry*s and *Timpi*s, *mtry* = 24 and *Timpi* = 5 (Timp5) generated the highest number of HACCs in pairwise comparison, 101 (averaged from three runs; 100, 101, 103 HACCs) out of 120 pairs (**Figure 1**). Although other combinations of *mtry*s and single-digit *Timpi*s resulted in similar numbers of HACCs, the run which contained the highest number of HACCs (103) was used exemplary for further analysis (**Figure 2**). The least number of HACCs (54) was computed from SVMs models using all available root traits (36). The number of HACCs thus increased by 91% through root trait selection with RF and tuning SVMs to Timp5. Both single SVMs model accuracy and the total number of HACCs based on six runs of five randomly selected root traits each (R_5.1–5.6) are similar or decreased compared to using all 36 root traits (All_36) and much lower than SVMs models involving the top five important root traits (Timp_5), respectively (**Figure 3**).

**FIGURE 4:**
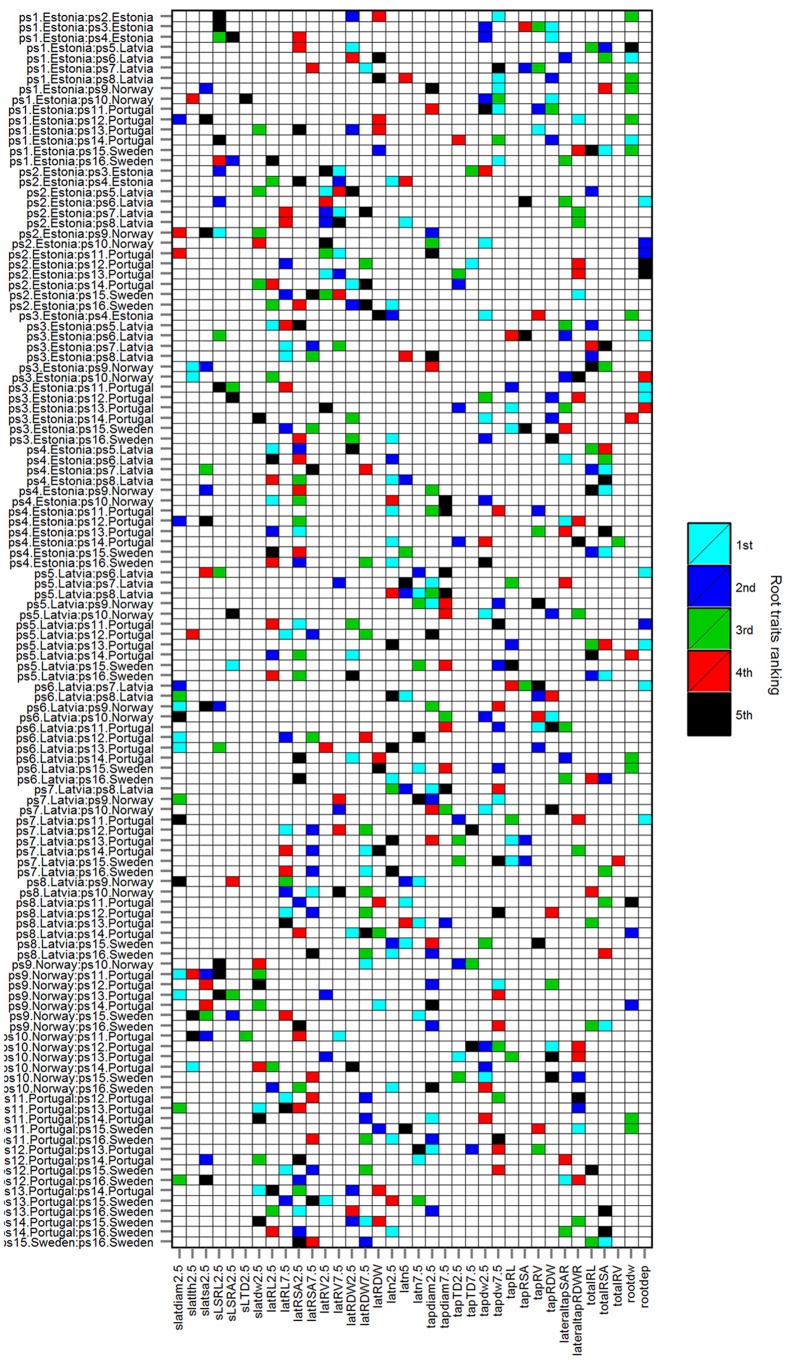
**Top five important root traits (Timp5) ranked as 1st, 2nd, 3rd, 4th, and 5^th^ in each of 103 HACCs; rbf SVMs prediction accuracy ≥ 80%.** Cultivars are listed in **Table 1**, root trait abbreviations in **Table 2**.

**FIGURE 5:**
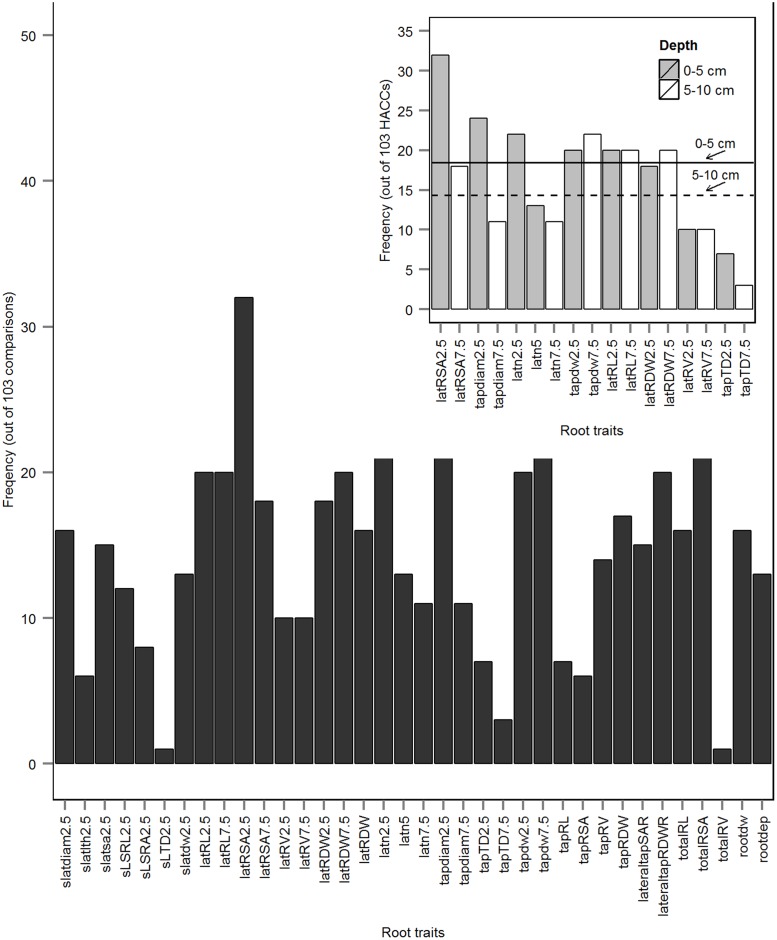
**Frequencies of top five important root traits (Timp5) in 103 high accuracy classifications (HACCs; rbf SVMs prediction accuracy ≥80%) identified out of 120 pairwise comparisons among 16 European *P. sativum* cultivars.** The inset illustrates the frequencies of root traits from 0 to 5 cm depth along the tap root (gray bars) and from 5 to 10 cm depth (white bars), respectively; a solid line indicates the average number of Timp5 from 0 to 5 cm depth, a dashed line indicates the average number of Timp5 from 5 to 10 cm depth tap root. Root trait abbreviations are listed in **Table 2**.

The Timp5 in each of the 103 HACCs are ranked as 1st, 2nd, 3rd, 4th, and 5th and indicated with different colors in **Figure 4** (see **Supplementary Table S1** for a list of Timp5 and **Supplementary Table S2** for normalized importance values of Timp5). The most frequent Timp5 of the analyzed pea cultivars in all HACCs (T5IRT) are latRSA2.5, tapdiam2.5, latn2.5, tapdw7.5, and totalRSA (see **Table 2** for trait abbreviations) with proportions of 31, 23, 21, 21, and 21%, respectively (**Figure 5**). The root traits measured at both 0–5 and 5–10 cm depth (along the tap root) are lateral root surface area, tap root diameter, lateral root number, tap root dry weight (tapRDW), lateral root length, lateral root dry weight (latRDW), lateral root volume, and tap root TD. Four out of eight root trait pairs measured at 0–5 cm depth (i.e., latRSA2.5, tapdiam2.5, latn2.5, latn5, and tapTD2.5) have a higher frequency among all Timp5 than corresponding ones from 5–10 cm depth along the tap root while the other four have similar frequencies (**Figure 5**, inset); the average frequency of Timp5 from 0–5 cm depth is 18 compared to 14 from 5–10 cm depth along the tap root.

**FIGURE 6:**
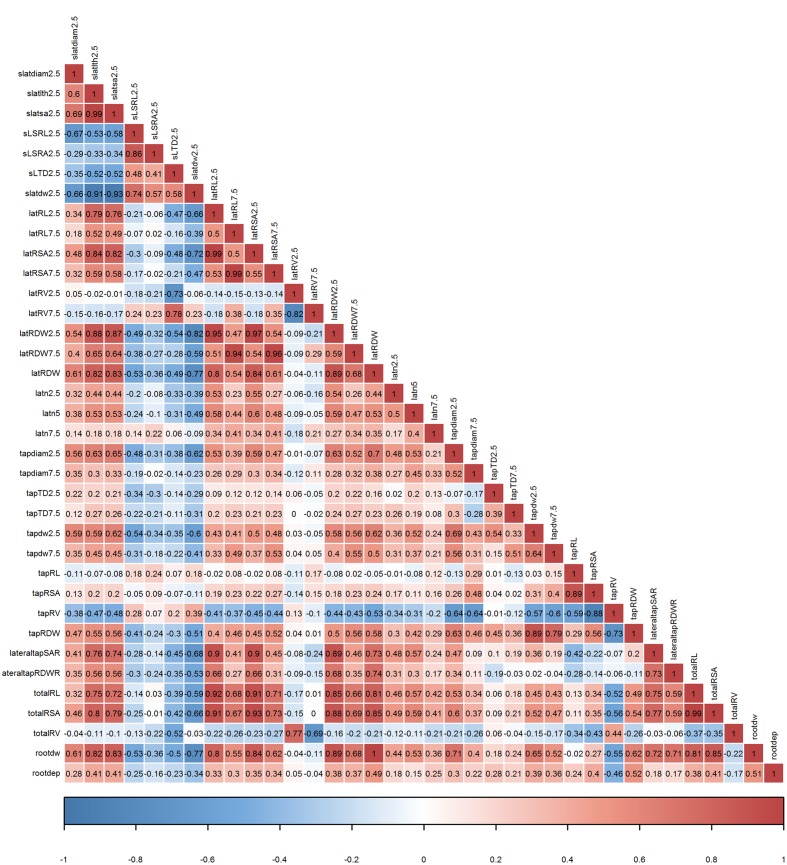
**Pearson correlation coefficients among 36 root traits determined on mature plants of 16 European *P. sativum* cultivars (*n* = 5–7).** Positive correlations are visualized by a red color gradient, negative correlations by a blue color gradient. Root trait abbreviations are listed in **Table 2**.

In order to confirm whether conditional inference permutation test can rank root traits without bias, correlation coefficients of Timp5s are compared in different pairwise classifications. For example, Timp5 in the pair ps1.Estonia vs. ps2.Estonia (**Figure 4**, first line; **Supplementary Tables S1** and **S2**) are tapRDW between 5–10 cm depth (tapdw7.5), lateral root try weight of 0–5 cm depth (latRDW2.5), total root system dry weight (rootdw), latRDW, and SRL of lateral roots at 0–5 cm depth (sSRL2.5). In this pair, the highest Pearson correlation between the most important root trait tapdw7.5 (ranked 1st) and the other four traits is 0.52 (**Figure 6**; see **Supplementary Figure S3** for Spearman correlation); tapRDW, which has a very high correlation coefficient of 0.89 with tapdw7.5, is not involved in the Timp5 of the pair ps1.Estonia vs. ps2.Estonia. Rootdw and latRDW, which are highly correlated with a Pearson coefficient of 1, ranked as 3rd and 4th in the classification of ps1.Estonia and ps2.Estonia (**Figure 6**). In another example (ps1.Estonia vs. ps7.Latvia), both rootdw and latRDW are among the Timp5 but rootdw is ranked 1st while latRDW is ranked 5th (**Figure 4**; **Supplementary Tables S1** and **S2**).

The highest correlation coefficient among T5IRTs was 0.93 (Pearson correlation, **Figure 6**; see **Supplementary Figure S3** for Spearman correlations) between latRSA2.5 and totalRSA while the lowest was 0.31 (Pearson) between tapdw7.5 and lateral root number at 0–2.5 cm depth (latn2.5). The frequencies of latRSA2.5 and totalRSA among T5IRTs (**Figure 5**) are among the highest with 32 and 22, respectively, while they only appear nine times simultaneously in the same pairwise classification.

### Permutation Test Comparing the Difference of the Mean of Single Root Trait

**FIGURE 7:**
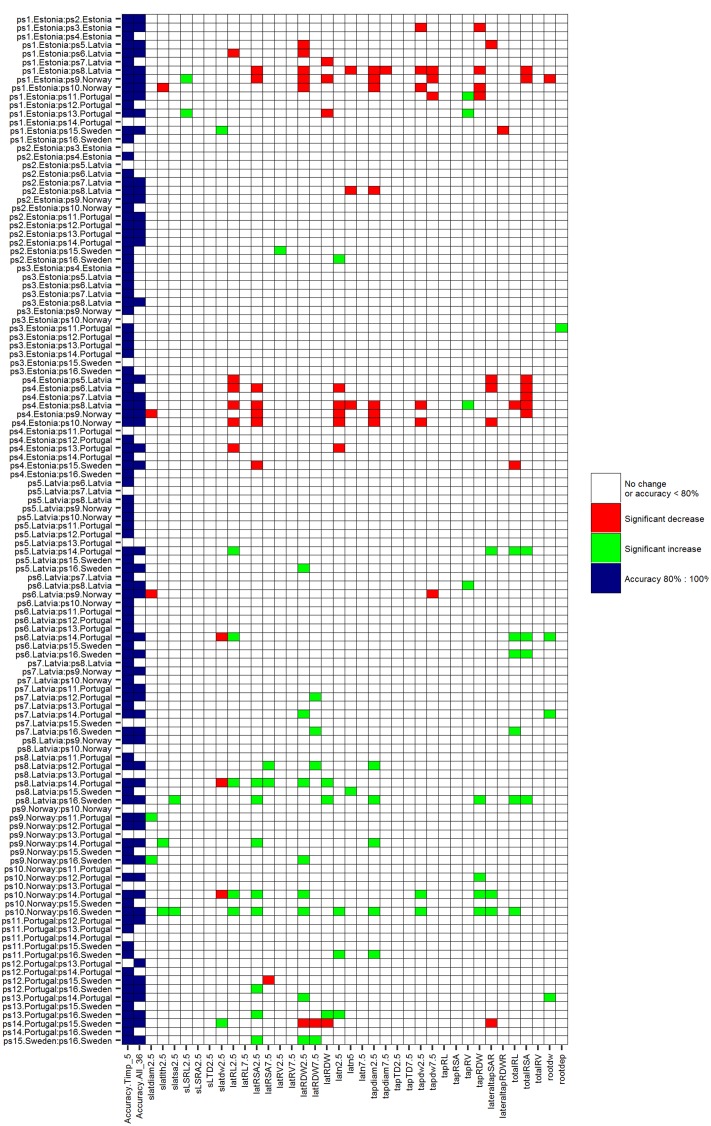
***P*-values of univariate permutation tests (Bonferroni-corrected) comparing the difference of means of each single root trait between 16 mature *P. sativum* L. cultivars in each pair.** Significantly (*P*-values < 0.05) increased root traits are marked green, significant decreased root traits are marked red, *P* ≥ 0.05 white; accuracy. Timp_5 and accuracy. All_36 (light blue) indicating HACCs with Timp5/all root traits involved in SVMs, respectively. Cultivars are listed in **Table 1**, root trait details in **Table 2**.

Comparing the efficiency of ML techniques conducted by RF and SVMs with a univariate permutation test, only 46.6% (Bonferroni-corrected) and 47.5% (fdr-corrected) of HACCs (based on Timp5) have significantly different root traits. Significantly different root traits in univariate permutation tests can be found in (supplementary) figures (**Figure 7**, with Bonferroni correction; **Supplementary Figure S4**, with fdr correction). SVMs were not always superior to univariate permutation test without root traits selection: classification accuracies of several pairwise SVMs classifications involving significantly different root traits in univariate permutation test were lower than 80% [see, e.g., ps11.Portugal vs. ps16.Sweden (**Figure 7**; **Supplementary Figure S4**)].

**FIGURE 8:**
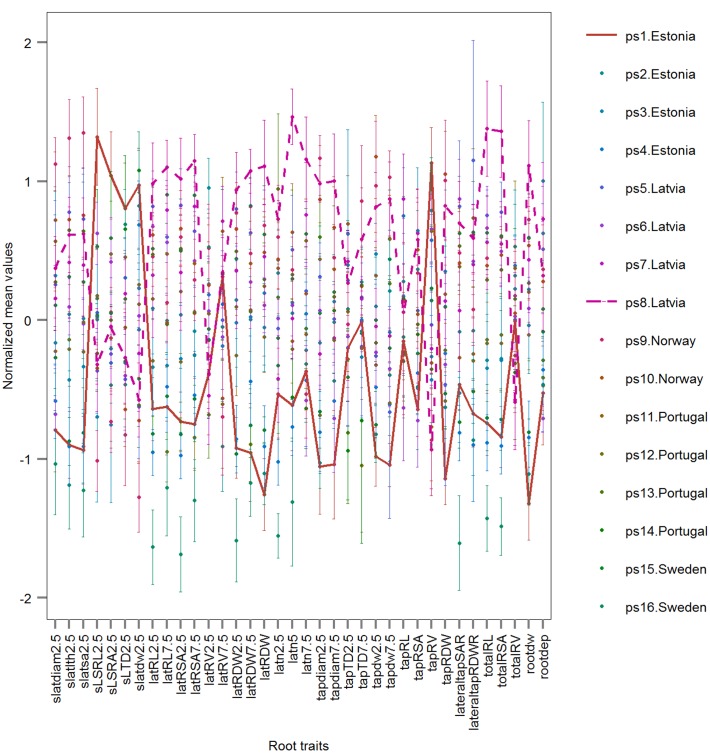
**Normalized root traits determined on mature plants of 16 European *P. sativum* cultivars (mean ± SE; *n* = 5–7).** Two horizontal lines visualize traits of the cultivars ps1.Estonia (solid) and ps8.Latvia (dashed). Cultivars are listed in **Table 1**, root trait abbreviations in **Table 2**.

Three examples are given to visualize the mean difference of root traits in pairwise comparisons. There are significant different root traits between ps1.Estonia vs. ps8.Latvia and the cultivars are also classified with high accuracy (92.3%; **Figure 8**). In contrast, cultivars ps3.Estonia vs. ps9.Norway are classified with high accuracy (90.9%), however, without any significant root traits identified by univariate permutation test (**Supplementary Figure S5**). There are (visibly) no significant different root traits between cultivars ps1.Estonia vs. ps14.Portugal and they are not accurately classified either – accuracy 69.2% (**Supplementary Figure S6**). The most significantly different root traits (the lowest *p* value in univariate permutation test) can be different from the most important root traits (ranked by RF) in each pairwise classification, e.g., Timp5 in the pair ps3.Estonia vs. ps9.Norway are tapdw7.5, tapRDW, rootdw, latn5, and latRDW while the rank order based on *p* values from permutation test changed to latn5, tapdw7.5, tapRDW, latRDW, and rootdw. No tied importance values of Timp5, indicating higher ranking root traits are more important in RF, have been found.

## Discussion

Machine learning algorithms are promising statistical tools to assist humans in the analysis of complex data sets and started to be widely used in many research fields including (plant) genomics/proteomics (Ma et al., 2014; Libbrecht and Noble, 2015). To the best of our knowledge, they have been only applied twice on RSA differentiation yet (Zhong et al., 2009; Iyer-Pascuzzi et al., 2010). This is surprising because as new technologies for generating large plant phenotypical data sets emerge, demand will drastically increase for new statistical techniques. Phenotyping is estimated in becoming the major operational bottleneck in limiting the power of genetic analysis and genomic prediction (Rahaman et al., 2015). Data complexity of particular sets of traits is generally high, especially in root systems where the developments causing a specific architecture, and the physiology and performance of individual root segments/units within the branched root system are not well understood yet.

So far, most of the techniques developed for RSA phenotyping involve the of use seedlings (Kuijken et al., 2015). Although there are examples in which the early stage root phenotype has predictive value for later developmental stages (Tuberosa et al., 2002), the seedling root phenotype may not always be representative of the mature plant (Watt et al., 2013). Because replicate numbers from manual phenotyping of mature root systems are limited but mature root systems often have a higher complexity, adapted statistical methods need to be developed to make full use of data sets. Here we applied combined ML algorithms with unbiased variable importance measure for the first time successfully, to the best of our knowledge, on a small RSA/root morphology data set manually derived from 97 mature plants of 16 European pea cultivars. The importance of 36 root traits was measured and ranked in RF. Pairwise classifications were analyzed either by SVMs based on a Gaussian radial basis kernel function (rbf SVMs) or by RF (standard algorithm) with the RF-identified top ranking root traits. The overall accuracy of models was cross-validated. The combination of SVMs and RF improved the classification accuracy – confirming earlier results by Löw et al. (2012) in remote sensing.

When compared to classical statistical tools, our results demonstrated that all pairwise classifications with significant root traits from univariate permutation test belong to HACCs with Timp5, however, almost half of HACCs derived from Timp5 don’t have any significantly different root traits. This points to the advantages of combining RF and SVMs in root traits importance measure and cultivar classification. Besides robustness to noises, RF considers both the influence of single variables separately and the multivariate interactions with other variables, which make this advanced ML approach more efficient, accurate, and reliable (Breiman, 2001; Zhu et al., 2012). Among the HACCs with significantly different root traits, the ranking of top five important root traits (Timp5) was not matched by *p* values from univariate permutation test, i.e., the most significantly different root traits differ with the most important root traits identified by pairwise classification. This might be due to that SVMs classification concerns more about parameters (root traits) involved in the SVMs models while traditional multi/univariate analyses focus more on differences of specific root traits between two groups (Gaonkar et al., 2015). We conclude that the combination of RF root traits selection and SVMs classification can make full use of all possible information of root traits in pea cultivars’ classification.

Our results clearly demonstrated the importance of selecting important root traits by RF to obtain an efficient classification based on RSA among dicot crop cultivars. SVMs models using all available traits or including five randomly selected root traits (R_5) were not able to increase the overall accuracy, which confirmed the necessity of root traits selection through RF in cultivar differentiation. This finding is in accordance with previous ML approaches in other scientific fields (Wang et al., 2010; Löw et al., 2012; Liu et al., 2014). The improved accuracy probably benefits from alleviating the ‘curse of dimensionality’ through root traits selection, removing non-informative signals (Chu et al., 2012). Thus, we can show that the identification of a few important root traits, in our case five, significantly increases the classification accuracy. While we did not find any single pea root trait that was always more important than others in all HACCs, a more targeted cultivar differentiation and trait selection for breeding can be obtained when focusing on root traits with highest frequency among T5IRTs. The most frequent T5IRT among the tested pea cultivars was the surface area of all lateral roots originating at the tap root between 0–5 cm (latRSA2.5) – appearing in more than one third of the pairwise comparisons. Distinguishing cultivars based on latRSA2.5 value can have important ecological effects: greater latRSA2.5 implicates more absorptive lateral root surface area in the topsoil and thus the potential for enhanced P or topsoil water foraging. Lower latRSA2.5 values means that plants are possibly privileging deep soil exploration (Miguel et al., 2015) with potential influences on drought tolerance or performance in low input agriculture (Bonser et al., 1996). Another frequent T5IRT in pea was latn2.5, the number of laterals originating at the tap root between 0–5 cm, which is somehow comparable to the trait ‘whorl numbers’ of *Phaseolus vulgaris* seedlings (Miguel et al., 2013). Miguel and colleagues could show that common bean genotypes with greater whorl numbers accumulated up to 60% more biomass under low-phosphorus conditions.

Completely intact root systems can hardly be collected by destructive harvesting methods, especially of mature plants with deeper root systems grown in the field; similar, lateral root traits are likely more affected by destructive sampling, e.g., by root tip shedding, than tap root traits. RSA information retrieved from top soil layers is thus likely more accurate (Miguel, 2012). Interestingly, the number of Timp5 derived from 0–5 cm depth of the pea tap root were more frequent than the ones originating from 5–10 cm depth, indicating that root traits from the top of the tap root have a greater potential to differentiate pea cultivars. This knowledge is already utilized by ‘shovelomics’ approaches, which only excavate the root crown of mature plants for phenotypical analysis (Trachsel et al., 2011; Bucksch et al., 2014). Our findings thus provide additional evidence that shovelomics can be considered an informative field-based high-throughput phenotyping approach due to the strong contribution of topsoil root traits to cultivar distinction.

Root system depth and average radius were previously identified as frequently top-ranked root traits in linear SVMs classification to distinguish different rice genotypes (Iyer-Pascuzzi et al., 2010). In our study the top two frequently important root traits were latRSA2.5 and tapdiam2.5 while root system depth (rootdep) was much less frequently present in Timp5. However, the top important root traits identified by Iyer-Pascuzzi et al. (2010) might be subject to change as the used ranking method was recently deemed biased (Gaonkar and Davatzikos, 2013). However, the difference of key root traits is likely also related to species-specific differences between rice and pea, but also the differences in growth stages (juvenile vs. mature), media (gel vs. sand), and analyzing methods (see “Discussion” above).

Correlation among traits is generally considered as an indication of their redundancy for classification. However, they may still provide complementary information and an otherwise inconclusive variable can provide a significant performance in combination with others (Guyon and Elisseeff, 2003). The correlation of Timp5 from all pairwise classifications varied greatly in this study: On the one hand, highly correlated root traits were not always top-ranked; on the other hand, root traits that were highly correlated with the most important Timp could even not be important at all in our study. The correlation variance of Timp5 thus confirms the unbiased root traits importance measure through conditional inference permutation test – increasing data interpretability.

## Conclusion

The accurate classification of 86% (103 of 120) genotype pairs of pea indicated that most of the studied cultivars could be well differentiated by using a few most distinguishing root traits, as selected through RF. This implies that past culturing did not lead to a major loss of RSA variability of the studied European pea cultivars. Breeders are envisioned to work more effectively in future breeding programs by knowing distinguishing (pea) root traits in advance (Manavalan et al., 2010). In specific, pairwise classification approaches can help breeders to make informed decisions on cultivars selection for crossing. Powerful statistical approaches are essential to make use of the increasing amount of phenotyping information available, integrating the complex trait sets. In particular, this study showed that combining RF with rbf SVMs for variable selection and group classification, respectively, can overcome problems in applying ML approaches on data sets characterized by a rather low signal-to-noise ratio. Thus, ML methods are generally envisioned to make plant phenotypical data analyses more effectively, robust and comprehensive. However, our experiment under standardized conditions might have caused the loss of root traits adaptive to local environmental conditions. Thus, further ML-supported analysis of field-derived root phenotypes under varying environments are urgently needed, selecting genotypes that feature specific sets of traits facilitating plant performance under local edaphic and climatic conditions. Advanced methods must be urgently developed in order to facilitate the phenotyping of mature root systems under realistic growing conditions.

## Author Contributions

JZ, GB, and BR conceived and planned the experiment; JZ and BR performed the experiment; JZ analyzed the data; all authors jointly wrote the manuscript.

## Conflict of Interest Statement

The authors declare that the research was conducted in the absence of any commercial or financial relationships that could be construed as a potential conflict of interest.
