# Supplementary material for: Phenotyping: Using Machine Learning for Improved Pairwise Genotype Classification Based on Root Traits
Source: Front Plant Sci. 2016 Dec 6;7:1864. doi: 10.3389/fpls.2016.01864 (PMC5138212; doi:10.3389/fpls.2016.01864)
Supplement: Supplementary file 1 [file Data_Sheet_1.docx]

## Supplementary material

Article title: **Phenotyping: Using machine learning for improved pair-wise genotype classification based on root traits**

Authors: Jiangsan Zhao, Gernot Bodner, Boris Rewald

The following Supplementary material is available for this article:

**Fig. S1** Multiclass classification of mature *Pisum sativum* L. cultivars with selected important root traits (imp.hacc) and with all 36 root traits (or.hacc).


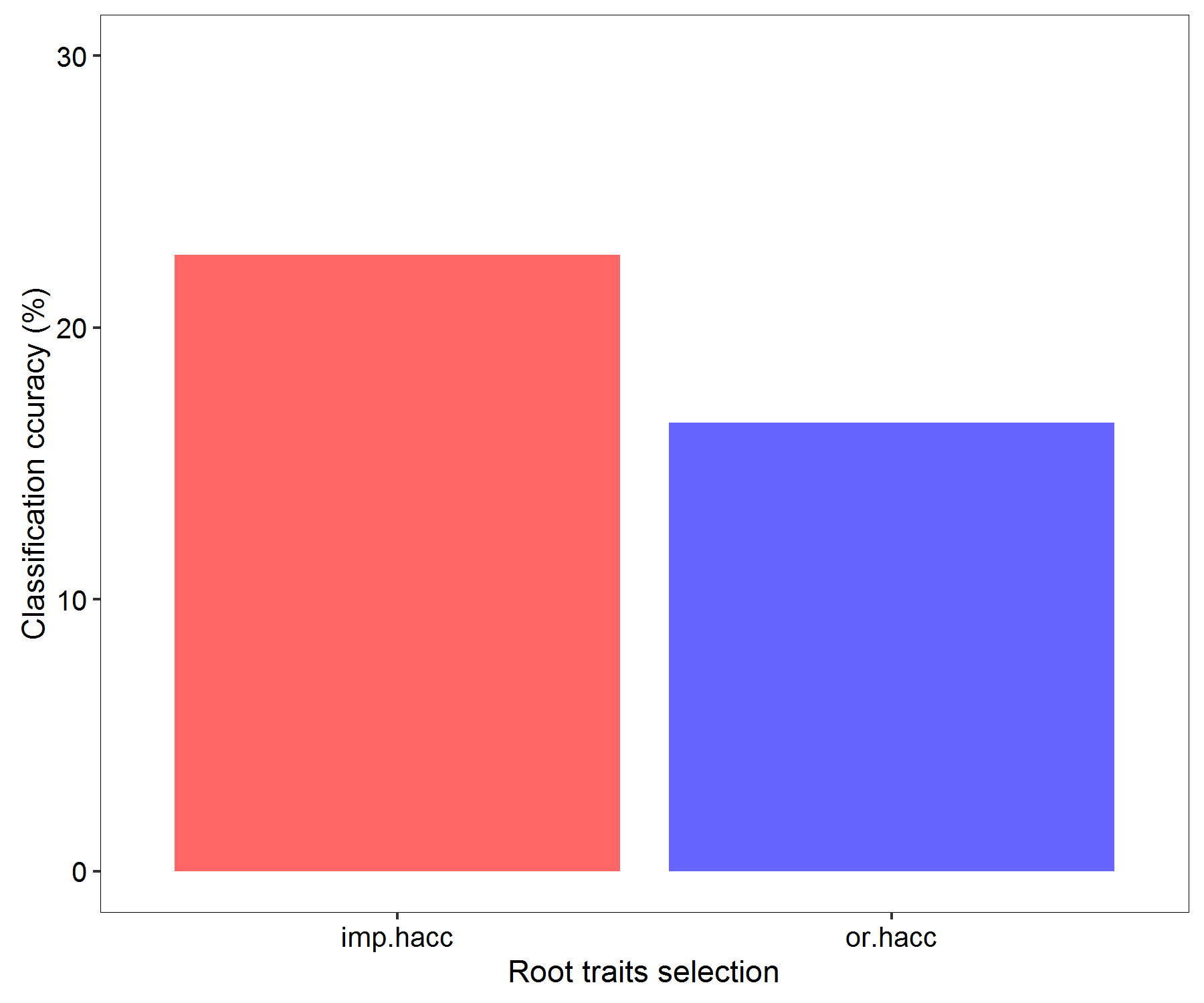


**Figure S1**

**Fig. S2** Comparison of the number of high accuracy classifications using RF models (HACCs; prediction accuracy of RF models ≥ 80%) with different numbers of *Pisum sativum* root traits randomly chosen for each tree, *mtry*s, and different number of top important root traits, *Timp_i*=1,2, 3, 5, 7, 9, 11, 13, 15 (mean±SE; n = 3 runs). 120 pairs of 16 European *Pisum sativum* cultivars were analyzed. A dashed blue line represents the number of HACCs (HACCN) retrieved using all 36 root traits in RF classification. A line connecting HACC numbers derived by using different number of *Timp*s at constant *mtry* = 32 was added for visualization.


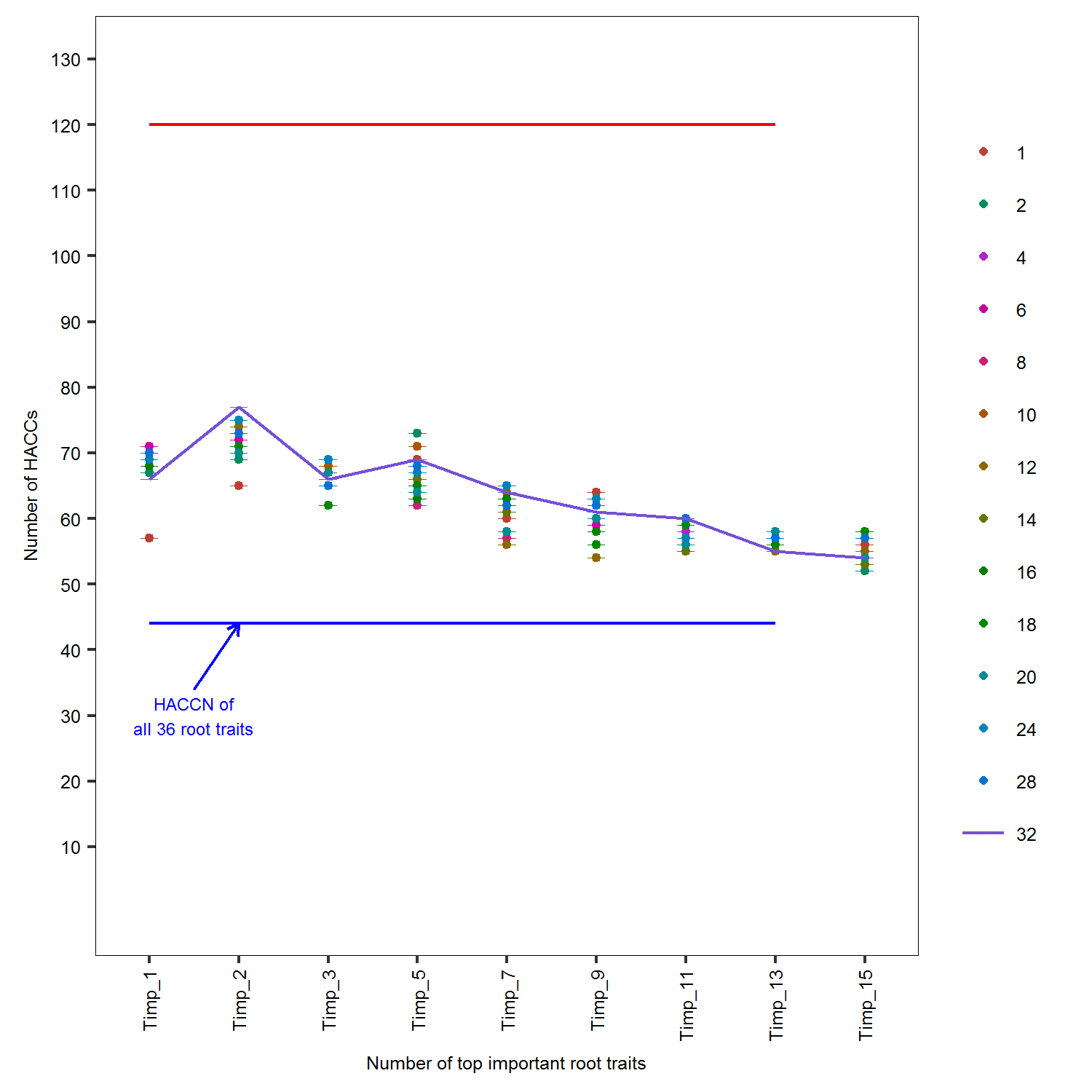


**Figure S2**

**Fig. S3** Spearman correlation of all 36 root traits. Values inside the cells are correlation coefficients. Root traits details are listed in Table 2.


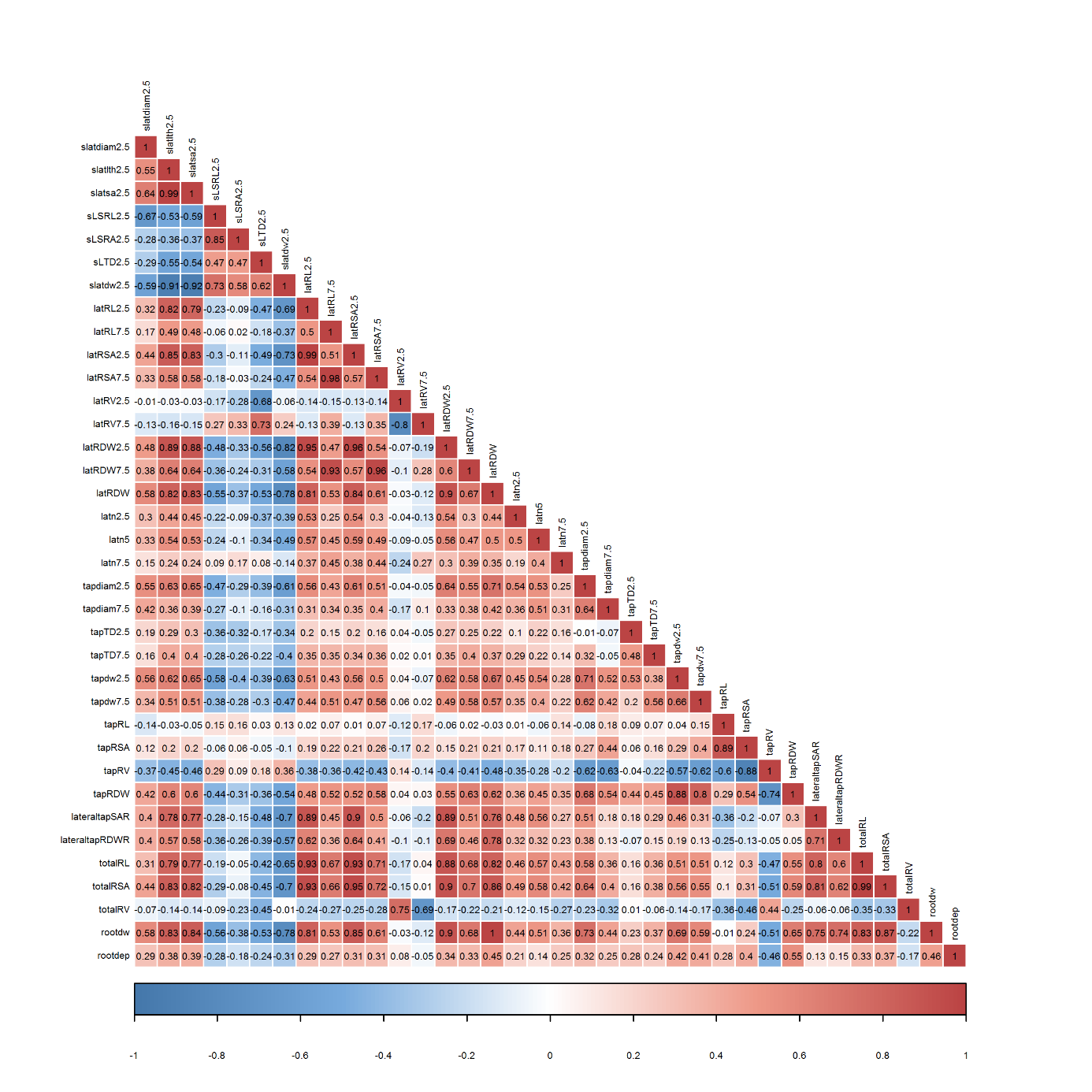


**Figure S3**

**Fig. S4** P values of univariate permutation (fdr correction) comparing the difference of means of each single root trait between 16 mature *Pisum sativum* L. cultivars in each pair. Significant (P <0.05) differences between root traits are marked green if increased in the second cultivar, red if significantly decreased; P≥0.05 are displayed as white; accuracy.Timp_5 and accuracy.All_36 (light blue) indicating HACCs with Timp5 / all root traits involved in SVMs respectively. Cultivars are listed in Table 1, root trait details in Table 2.


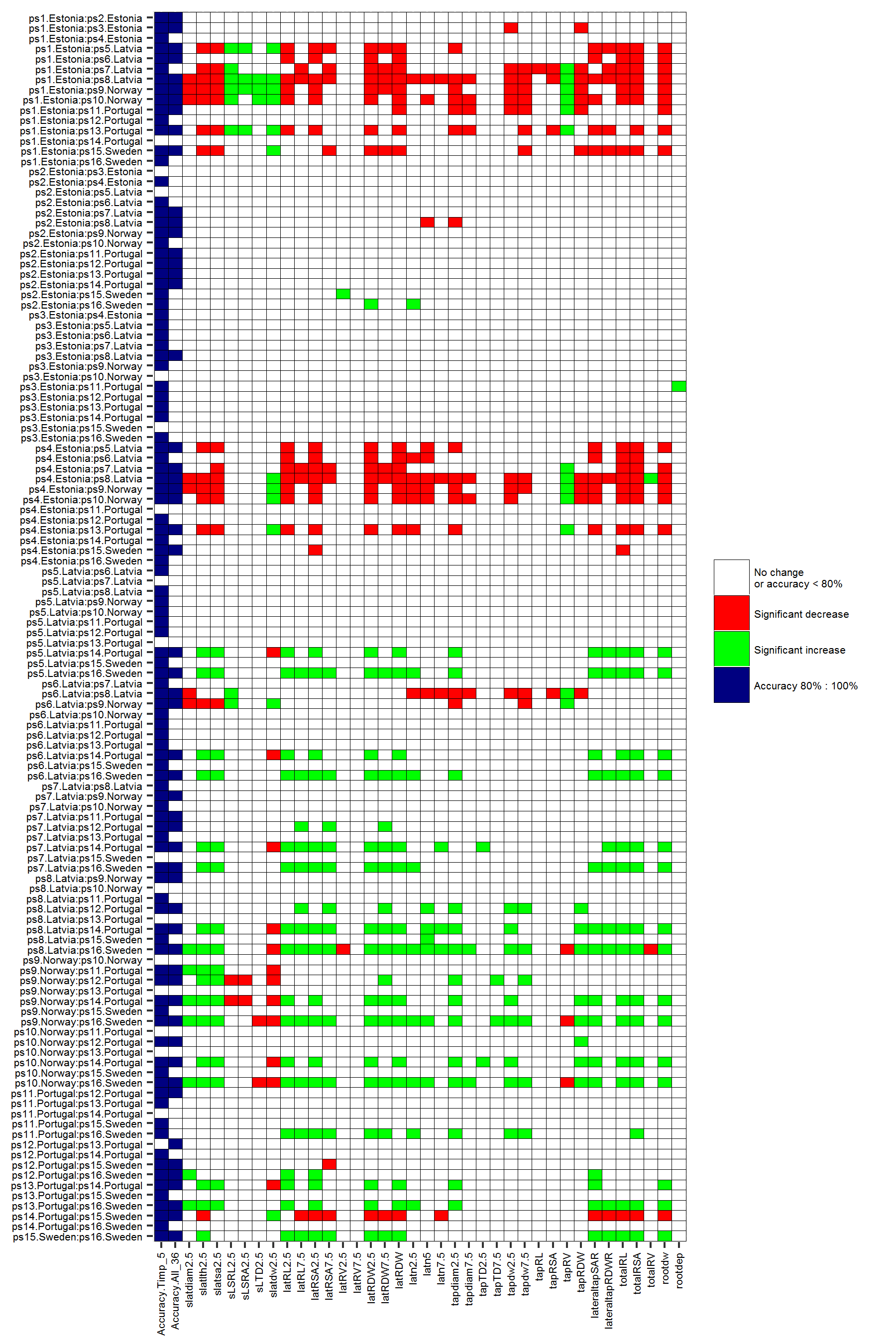


**Figure S4**

**Fig. S5** Root traits differentiation of 16 pea cultivars. Normalized mean values of all root traits were examined. The points represent means of normalized values, and the small lines represent SEs. The two horizontal lines are for better visualization of ps3.Estonia and ps9.Norway. Cultivar details in each pairwise comparison are shown in Table 1; root trait details are listed in Table 2.


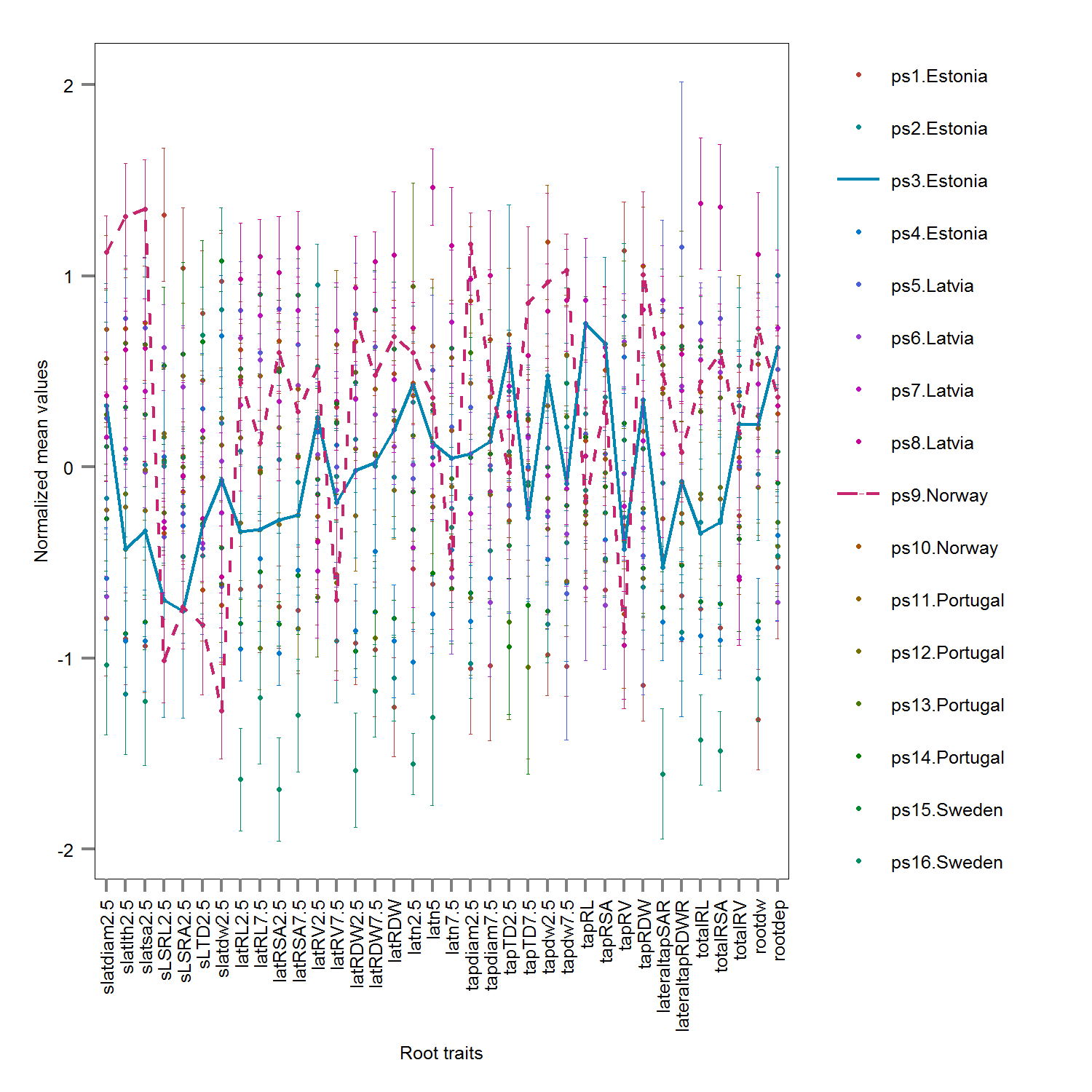


**Figure S5**

**Fig. S6** Root traits differentiation of 16 pea cultivars. Normalized mean values of all root traits were examined. The points represent means of normalized values, and the small lines represent SEs. The two horizontal lines are for better visualization of ps1.Estonia and ps14.Portugal. Cultivar details in each pairwise comparison are shown in Table 1; root trait details are listed in Table 2.


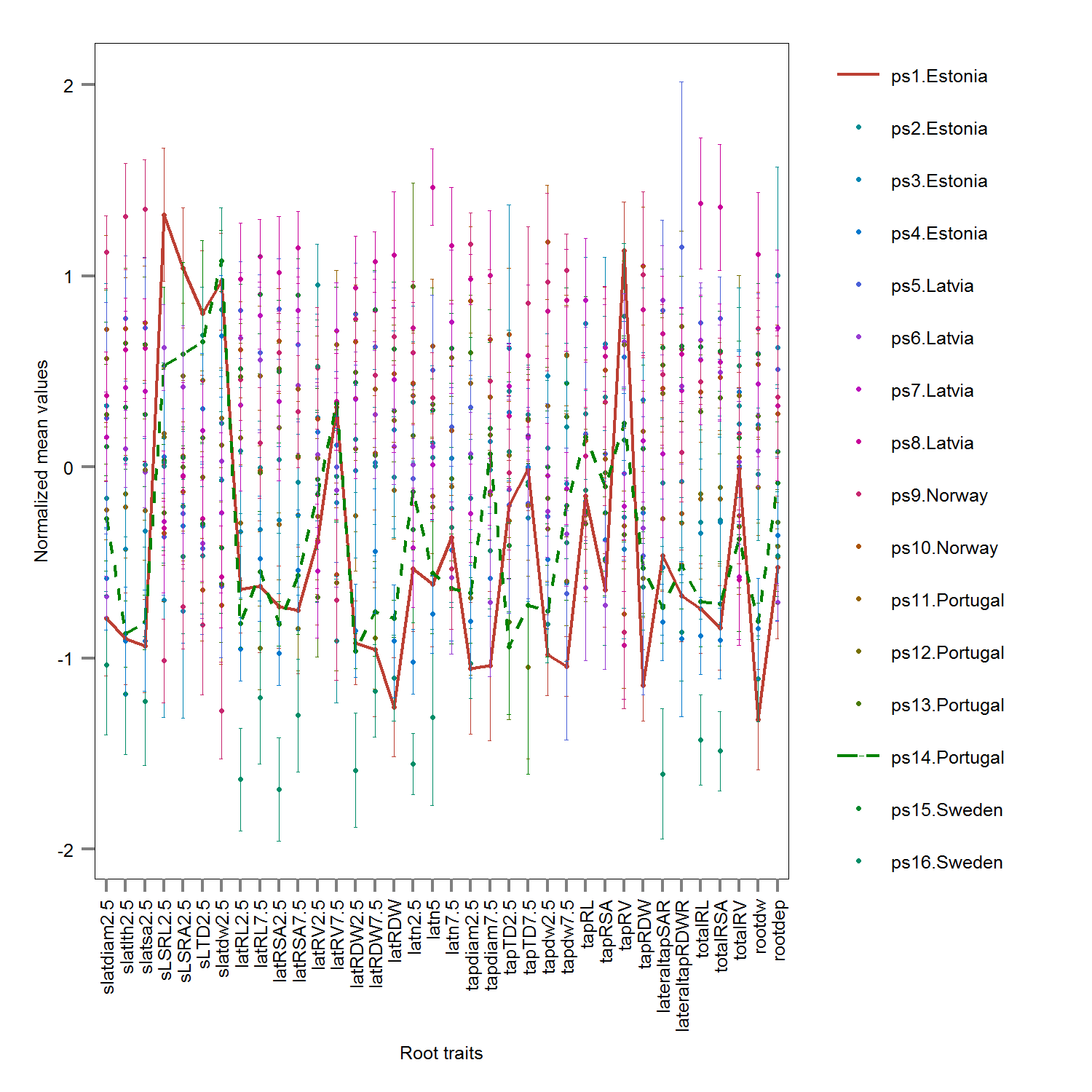


**Figure S6**

**Table S1** Top 5 important root traits in each pairwise classification of 16 pea cultivars. Rank 1st, 2nd, 3rd, 4th and 5th means the most important to the 5th important root trait in each pairwise classification. Cultivar details in each pairwise comparison are shown in Table 1; root trait details are listed in Table 2.

| Pairwise comparison | Rank 1st | Rank 2nd | Rank 3rd | Rank 4th | Rank 5th |
| --- | --- | --- | --- | --- | --- |
| ps1.Estonia:ps2.Estonia | tapdw7.5 | latRDW2.5 | rootdw | latRDW | sSRL2.5 |
| ps1.Estonia:ps3.Estonia | tapRDW | tapdw2.5 | tapRV | tapRSA | sSRL2.5 |
| ps1.Estonia:ps4.Estonia | tapRDW | tapdw2.5 | sSRL2.5 | latRSA2.5 | sLSRA2.5 |
| ps1.Estonia:ps5.Latvia | latRDW2.5 | totalRSA | totalRL | latRSA2.5 | rootdw |
| ps1.Estonia:ps6.Latvia | rootdw | lateraltapSAR | totalRSA | latRDW2.5 | latRDW |
| ps1.Estonia:ps7.Latvia | latRDW7.5 | tapRSA | tapRV | latRSA7.5 | tapdw7.5 |
| ps1.Estonia:ps8.Latvia | tapdw7.5 | tapRDW | rootdw | latn5 | latRDW |
| ps1.Estonia:ps9.Norway | tapdw7.5 | slatsa2.5 | rootdw | totalRSA | tapdiam2.5 |
| ps1.Estonia:ps10.Norway | tapRDW | tapdw2.5 | tapdw7.5 | slatlth2.5 | sLTD2.5 |
| ps1.Estonia:ps11.Portugal | tapdw7.5 | tapRV | tapRDW | tapdiam2.5 | tapdw2.5 |
| ps1.Estonia:ps12.Portugal | lateraltapRDWR | slatdiam2.5 | rootdw | latRDW | slatsa2.5 |
| ps1.Estonia:ps13.Portugal | tapRV | latRDW2.5 | slatdw2.5 | latRDW | latRSA2.5 |
| ps1.Estonia:ps15.Sweden | totalRSA | latRDW | rootdw | lateraltapRDWR | totalRL |
| ps1.Estonia:ps16.Sweden | tapdw7.5 | sLSRA2.5 | lateraltapSAR | sSRL2.5 | latRL2.5 |
| ps2.Estonia:ps4.Estonia | latn2.5 | latRV7.5 | latRL2.5 | latn5 | latRSA2.5 |
| ps2.Estonia:ps6.Latvia | rootdep | sSRL2.5 | lateraltapSAR | latRV2.5 | tapRSA |
| ps2.Estonia:ps7.Latvia | latRV7.5 | latRV2.5 | lateraltapRDWR | latRL7.5 | latRDW7.5 |
| ps2.Estonia:ps8.Latvia | latn5 | latRV2.5 | lateraltapRDWR | latRL7.5 | latRV7.5 |
| ps2.Estonia:ps9.Norway | sSRL2.5 | tapdiam2.5 | slatdw2.5 | slatdiam2.5 | slatsa2.5 |
| ps2.Estonia:ps10.Norway | tapdw2.5 | rootdep | tapdiam2.5 | slatdw2.5 | latRV2.5 |
| ps2.Estonia:ps11.Portugal | latRV7.5 | rootdep | latRV2.5 | slatdiam2.5 | tapdiam2.5 |
| ps2.Estonia:ps12.Portugal | tapTD7.5 | latRL7.5 | latRDW7.5 | lateraltapRDWR | rootdep |
| ps2.Estonia:ps13.Portugal | latRV2.5 | latRV7.5 | tapTD2.5 | lateraltapRDWR | rootdep |
| ps2.Estonia:ps14.Portugal | latRDW2.5 | tapTD2.5 | slatdw2.5 | latRL2.5 | latRDW7.5 |
| ps2.Estonia:ps15.Sweden | lateraltapRDWR | latRL7.5 | latRV2.5 | latRV7.5 | latRSA7.5 |
| ps2.Estonia:ps16.Sweden | latn2.5 | latRDW2.5 | latRL2.5 | latRSA2.5 | latRDW7.5 |
| ps3.Estonia:ps4.Estonia | tapdw2.5 | latn2.5 | rootdw | tapRV | latRDW |
| ps3.Estonia:ps5.Latvia | latRL2.5 | totalRL | lateraltapSAR | latRL7.5 | latRSA2.5 |
| ps3.Estonia:ps6.Latvia | rootdep | lateraltapSAR | sSRL2.5 | tapRL | tapRSA |
| ps3.Estonia:ps7.Latvia | latRL7.5 | latRSA7.5 | latRV7.5 | totalRL | totalRSA |
| ps3.Estonia:ps8.Latvia | latRL7.5 | totalRL | latRSA7.5 | latn5 | tapdiam2.5 |
| ps3.Estonia:ps9.Norway | slatlth2.5 | slatsa2.5 | totalRSA | tapdiam2.5 | totalRL |
| ps3.Estonia:ps11.Portugal | rootdep | tapRL | sLSRA2.5 | latRL7.5 | sSRL2.5 |
| ps3.Estonia:ps12.Portugal | rootdep | tapRDW | tapdw2.5 | lateraltapRDWR | sLSRA2.5 |
| ps3.Estonia:ps13.Portugal | tapRL | tapTD2.5 | lateraltapSAR | rootdep | latRV2.5 |
| ps3.Estonia:ps14.Portugal | tapdw2.5 | tapRDW | latRDW2.5 | rootdw | slatdw2.5 |
| ps3.Estonia:ps16.Sweden | latn2.5 | tapdw2.5 | latRDW2.5 | latRSA2.5 | tapRDW |
| ps4.Estonia:ps5.Latvia | latRL2.5 | latRSA2.5 | totalRL | totalRSA | latRDW2.5 |
| ps4.Estonia:ps6.Latvia | lateraltapSAR | latn2.5 | totalRSA | latRSA2.5 | latRL2.5 |
| ps4.Estonia:ps7.Latvia | totalRSA | totalRL | slatsa2.5 | latRDW7.5 | latRSA7.5 |
| ps4.Estonia:ps8.Latvia | latn2.5 | latn5 | latRSA2.5 | latRL2.5 | totalRSA |
| ps4.Estonia:ps9.Norway | totalRSA | slatsa2.5 | tapdiam2.5 | latRSA2.5 | totalRL |
| ps4.Estonia:ps10.Norway | latRL2.5 | tapdw2.5 | latRSA2.5 | latn2.5 | tapdiam7.5 |

**Table S1 (continued)**

| Pairwise comparison | Rank 1st | Rank 2nd | Rank 3rd | Rank 4th | Rank 5th |
| --- | --- | --- | --- | --- | --- |
| ps4.Estonia:ps12.Portugal | lateraltapSAR | slatdiam2.5 | latRSA2.5 | lateraltapRDWR | slatsa2.5 |
| ps4.Estonia:ps13.Portugal | latRSA2.5 | latRL2.5 | tapRV | lateraltapSAR | totalRSA |
| ps4.Estonia:ps14.Portugal | latn2.5 | tapTD2.5 | totalRV | tapdw2.5 | lateraltapRDWR |
| ps4.Estonia:ps15.Sweden | totalRSA | totalRL | latn5 | latRSA2.5 | latRL2.5 |
| ps4.Estonia:ps16.Sweden | latn2.5 | latRSA2.5 | latRDW7.5 | latRL2.5 | tapdw2.5 |
| ps5.Latvia:ps6.Latvia | rootdep | latn7.5 | sSRL2.5 | slatsa2.5 | tapdiam7.5 |
| ps5.Latvia:ps8.Latvia | latn7.5 | latn5 | tapdiam2.5 | latn2.5 | tapdiam7.5 |
| ps5.Latvia:ps9.Norway | tapdiam2.5 | tapdw7.5 | latn7.5 | tapdiam7.5 | tapRV |
| ps5.Latvia:ps10.Norway | tapdw2.5 | tapRDW | lateraltapRDWR | tapdiam7.5 | sLSRA2.5 |
| ps5.Latvia:ps11.Portugal | latRSA2.5 | rootdep | latRDW2.5 | latRL2.5 | tapdw7.5 |
| ps5.Latvia:ps12.Portugal | latRL7.5 | latRSA7.5 | latRDW7.5 | slatlth2.5 | tapdiam2.5 |
| ps5.Latvia:ps14.Portugal | latRDW2.5 | latRL2.5 | latRSA2.5 | rootdw | totalRL |
| ps5.Latvia:ps15.Sweden | sLSRA2.5 | tapdw7.5 | latn7.5 | tapdiam7.5 | tapRL |
| ps5.Latvia:ps16.Sweden | totalRSA | totalRL | latRSA2.5 | latRL2.5 | latRDW2.5 |
| ps6.Latvia:ps7.Latvia | rootdep | slatdiam2.5 | tapRSA | tapRL | tapRV |
| ps6.Latvia:ps8.Latvia | latn5 | tapRV | slatdiam2.5 | tapRDW | latn2.5 |
| ps6.Latvia:ps9.Norway | slatdiam2.5 | sSRL2.5 | tapdiam2.5 | tapdw7.5 | slatsa2.5 |
| ps6.Latvia:ps10.Norway | tapRDW | tapdw2.5 | tapdiam7.5 | tapRV | slatdiam2.5 |
| ps6.Latvia:ps11.Portugal | tapRV | tapdw7.5 | lateraltapSAR | tapdiam7.5 | tapRDW |
| ps6.Latvia:ps12.Portugal | slatdiam2.5 | latRL7.5 | latRSA7.5 | latRDW7.5 | latn7.5 |
| ps6.Latvia:ps13.Portugal | slatdiam2.5 | tapRV | sSRL2.5 | latRV2.5 | latn2.5 |
| ps6.Latvia:ps14.Portugal | latRDW2.5 | lateraltapSAR | rootdw | latRDW | latRSA2.5 |
| ps6.Latvia:ps15.Sweden | latn7.5 | tapdw7.5 | rootdw | tapdiam7.5 | latRDW |
| ps6.Latvia:ps16.Sweden | latn2.5 | totalRSA | lateraltapSAR | totalRL | latRSA2.5 |
| ps7.Latvia:ps8.Latvia | tapdiam2.5 | latn5 | latn2.5 | tapdw7.5 | tapdiam7.5 |
| ps7.Latvia:ps9.Norway | tapdw7.5 | tapdiam2.5 | slatdiam2.5 | latRV7.5 | latn7.5 |
| ps7.Latvia:ps10.Norway | tapdw2.5 | latRV7.5 | tapdiam7.5 | tapdiam2.5 | tapRDW |
| ps7.Latvia:ps11.Portugal | rootdep | tapTD2.5 | tapRL | lateraltapRDWR | slatdiam2.5 |
| ps7.Latvia:ps12.Portugal | latRL7.5 | latRSA7.5 | latRDW7.5 | latRV7.5 | tapTD7.5 |
| ps7.Latvia:ps13.Portugal | tapRL | tapRSA | tapTD2.5 | tapdiam2.5 | latn2.5 |
| ps7.Latvia:ps14.Portugal | latRDW7.5 | latRSA7.5 | lateraltapRDWR | latRL7.5 | latRDW |
| ps7.Latvia:ps16.Sweden | latRDW7.5 | latRSA7.5 | totalRSA | latRL7.5 | latn2.5 |
| ps8.Latvia:ps9.Norway | latn7.5 | latn5 | latRL7.5 | sLSRA2.5 | slatdiam2.5 |
| ps8.Latvia:ps11.Portugal | latn5 | latRSA2.5 | totalRSA | latRDW | rootdw |
| ps8.Latvia:ps12.Portugal | latRL7.5 | latRSA7.5 | latRDW7.5 | tapRDW | tapdw7.5 |
| ps8.Latvia:ps14.Portugal | latRDW2.5 | rootdw | latRDW | latRSA2.5 | latRDW7.5 |
| ps8.Latvia:ps15.Sweden | latn5 | latn2.5 | tapdw2.5 | tapdiam2.5 | tapRV |
| ps8.Latvia:ps16.Sweden | latn2.5 | tapdiam2.5 | latRDW7.5 | totalRSA | latRSA7.5 |
| ps9.Norway:ps11.Portugal | slatdiam2.5 | slatsa2.5 | slatdw2.5 | slatlth2.5 | sSRL2.5 |
| ps9.Norway:ps12.Portugal | tapdw7.5 | tapdiam2.5 | tapRDW | slatsa2.5 | slatdw2.5 |
| ps9.Norway:ps14.Portugal | latRDW | rootdw | slatdw2.5 | slatsa2.5 | tapdiam2.5 |
| ps9.Norway:ps15.Sweden | latn7.5 | sLSRA2.5 | slatsa2.5 | latRL7.5 | slatlth2.5 |
| s9.Norway:ps16.Sweden | totalRSA | tapdiam2.5 | totalRL | tapdw7.5 | latRSA2.5 |
| ps10.Norway:ps12.Portugal | tapRDW | tapdw2.5 | tapdw7.5 | lateraltapRDWR | tapTD7.5 |
| ps10.Norway:ps14.Portugal | slatlth2.5 | tapdw2.5 | latRL2.5 | slatdw2.5 | latRDW2.5 |

**Table S1 (continued)**

| Pairwise comparison | Rank 1st | Rank 2nd | Rank 3rd | Rank 4th | Rank 5th |
| --- | --- | --- | --- | --- | --- |
| ps10.Norway:ps15.Sweden | tapdw2.5 | lateraltapRDWR | tapTD2.5 | latRSA7.5 | tapRDW |
| ps10.Norway:ps16.Sweden | latn2.5 | latRL2.5 | latRSA2.5 | tapdw2.5 | tapdiam2.5 |
| ps11.Portugal:ps12.Portugal | latRL7.5 | latRDW7.5 | tapdw7.5 | latRSA7.5 | lateraltapRDWR |
| ps11.Portugal:ps13.Portugal | slatdw2.5 | lateraltapRDWR | slatdiam2.5 | latRSA2.5 | latRL7.5 |
| ps11.Portugal:ps15.Sweden | lateraltapRDWR | latRDW | rootdw | tapRV | latn5 |
| ps11.Portugal:ps16.Sweden | latn2.5 | tapdiam2.5 | latRDW7.5 | latRSA7.5 | tapdw7.5 |
| ps12.Portugal:ps14.Portugal | latn7.5 | slatsa2.5 | slatdw2.5 | lateraltapSAR | latRSA2.5 |
| ps12.Portugal:ps15.Sweden | latRL7.5 | latRSA7.5 | latRDW7.5 | tapdw7.5 | totalRL |
| ps12.Portugal:ps16.Sweden | lateraltapSAR | latRSA2.5 | slatdiam2.5 | lateraltapRDWR | slatsa2.5 |
| ps13.Portugal:ps14.Portugal | slatdw2.5 | latRDW2.5 | latRSA2.5 | latRDW | latRL2.5 |
| ps13.Portugal:ps15.Sweden | latRV2.5 | latRL7.5 | latn7.5 | latn2.5 | latRSA7.5 |
| ps13.Portugal:ps16.Sweden | latRSA2.5 | tapdiam2.5 | latRL2.5 | latRDW2.5 | totalRSA |
| ps14.Portugal:ps15.Sweden | latRDW7.5 | latRDW2.5 | lateraltapRDWR | latRDW | slatdw2.5 |
| ps14.Portugal:ps16.Sweden | latn2.5 | latRSA2.5 | lateraltapSAR | latRL2.5 | totalRSA |
| ps15.Sweden:ps16.Sweden | totalRSA | latRDW7.5 | totalRL | latRSA7.5 | latRSA2.5 |

**Table S2** Importance values of top 5 important root traits in each pairwise classification of 16 pea cultivars. Cultivar details are shown in Table 1. The values are normalized between 0 and 1.

| Pairwise comparison | Rank 1st | Rank 2nd | Rank 3rd | Rank 4th | Rank 5th |
| --- | --- | --- | --- | --- | --- |
| ps1.Estonia:ps2.Estonia | 1 | 0.051133 | 0.022333 | 0.015667 | 0.013533 |
| ps1.Estonia:ps3.Estonia | 1 | 0.0768 | 0.058489 | 0.002222 | 0.002178 |
| ps1.Estonia:ps4.Estonia | 1 | 0.034471 | 0.017147 | 0.008191 | 0.0045 |
| ps1.Estonia:ps5.Latvia | 1 | 0.028747 | 0.027653 | 0.004453 | 0.003413 |
| ps1.Estonia:ps6.Latvia | 1 | 0.037125 | 0.01 | 0.006833 | 0.005896 |
| ps1.Estonia:ps7.Latvia | 1 | 0.019821 | 0.018107 | 0.006804 | 0.004518 |
| ps1.Estonia:ps8.Latvia | 1 | 0.023328 | 0.022344 | 0.003859 | 0.0015 |
| ps1.Estonia:ps9.Norway | 1 | 0.033194 | 0.008236 | 0.005264 | 0.001125 |
| ps1.Estonia:ps10.Norway | 1 | 0.024744 | 0.018633 | 0.001011 | 0.000778 |
| ps1.Estonia:ps11.Portugal | 1 | 0.020222 | 0.007061 | 0.00501 | 0.000343 |
| ps1.Estonia:ps12.Portugal | 1 | 0.009021 | 0.002938 | 0.001958 | 0.001875 |
| ps1.Estonia:ps13.Portugal | 1 | 0.015808 | 0.006644 | 0.004346 | 0.002875 |
| ps1.Estonia:ps15.Sweden | 1 | 0.011067 | 0.005128 | 0.001826 | 0.001518 |
| ps1.Estonia:ps16.Sweden | 1 | 0.010609 | 0.003063 | 0.001477 | 0.00125 |
| ps2.Estonia:ps4.Estonia | 1 | 0.056133 | 0.010433 | 0.0103 | 0.002867 |
| ps2.Estonia:ps6.Latvia | 1 | 0.029667 | 0.009385 | 0.004538 | 0.002821 |
| ps2.Estonia:ps7.Latvia | 1 | 0.041407 | 0.010462 | 0.002593 | 0.001473 |
| ps2.Estonia:ps8.Latvia | 1 | 0.035615 | 0.012962 | 0.004038 | 0.001538 |
| ps2.Estonia:ps9.Norway | 1 | 0.019658 | 0.010974 | 0.010769 | 0.00053 |
| ps2.Estonia:ps10.Norway | 1 | 0.01305 | 0.00295 | 0.002438 | 0.00185 |
| ps2.Estonia:ps11.Portugal | 1 | 0.013182 | 0.005852 | 0.002864 | 0.001057 |
| ps2.Estonia:ps12.Portugal | 1 | 0.008423 | 0.004577 | 0.003269 | 0.002192 |
| ps2.Estonia:ps13.Portugal | 1 | 0.018272 | 0.001467 | 0.001148 | 0.001136 |
| ps2.Estonia:ps14.Portugal | 1 | 0.021033 | 0.00289 | 0.001824 | 0.001626 |
| ps2.Estonia:ps15.Sweden | 1 | 0.020582 | 0.003406 | 0.002279 | 0.000739 |
| ps2.Estonia:ps16.Sweden | 1 | 0.019221 | 0.005192 | 0.00249 | 0.000356 |
| ps3.Estonia:ps4.Estonia | 1 | 0.046467 | 0.029933 | 0.0014 | 9.00E-04 |
| ps3.Estonia:ps5.Latvia | 1 | 0.030367 | 0.028833 | 0.004267 | 0.0039 |
| ps3.Estonia:ps6.Latvia | 1 | 0.051615 | 0.007051 | 0.004128 | 0.001923 |
| ps3.Estonia:ps7.Latvia | 1 | 0.037692 | 0.013099 | 0.002308 | 0.000879 |
| ps3.Estonia:ps8.Latvia | 1 | 0.022115 | 0.011385 | 0.00475 | 0.002173 |
| ps3.Estonia:ps9.Norway | 1 | 0.022786 | 0.007094 | 0.003949 | 0.000752 |
| ps3.Estonia:ps11.Portugal | 1 | 0.020648 | 0.001773 | 0.001261 | 0.00033 |
| ps3.Estonia:ps12.Portugal | 1 | 0.015256 | 0.004 | 0.000936 | 0.000821 |
| ps3.Estonia:ps13.Portugal | 1 | 0.009964 | 0.004604 | 0.002355 | 0.001124 |
| ps3.Estonia:ps14.Portugal | 1 | 0.016967 | 0.002747 | 0.002 | 0.001121 |
| ps3.Estonia:ps16.Sweden | 1 | 0.018779 | 0.005115 | 0.000663 | 0.000596 |
| ps4.Estonia:ps5.Latvia | 1 | 0.029867 | 0.02448 | 0.0224 | 0.011333 |
| ps4.Estonia:ps6.Latvia | 1 | 0.034375 | 0.011667 | 0.009958 | 0.009104 |
| ps4.Estonia:ps7.Latvia | 1 | 0.041161 | 0.011839 | 0.002821 | 0.001393 |
| ps4.Estonia:ps8.Latvia | 1 | 0.02925 | 0.021703 | 0.003797 | 0.002281 |
| ps4.Estonia:ps9.Norway | 1 | 0.029236 | 0.007431 | 0.005861 | 0.002528 |
| ps4.Estonia:ps10.Norway | 1 | 0.012889 | 0.010733 | 0.0058 | 0.004111 |

**Table S2 (continued)**

| Pairwise comparison | Rank 1st | Rank 2nd | Rank 3rd | Rank 4th | Rank 5th |
| --- | --- | --- | --- | --- | --- |
| ps4.Estonia:ps12.Portugal | 1 | 0.009745 | 0.004615 | 0.00387 | 0.00126 |
| ps4.Estonia:ps13.Portugal | 1 | 0.017856 | 0.005144 | 0.001433 | 0.000981 |
| ps4.Estonia:ps14.Portugal | 1 | 0.009866 | 0.00896 | 0.001143 | 0.000196 |
| ps4.Estonia:ps15.Sweden | 1 | 0.018933 | 0.006154 | 0.002308 | 0.000349 |
| ps4.Estonia:ps16.Sweden | 1 | 0.003391 | 0.002555 | 0.002328 | 0.001734 |
| ps5.Latvia:ps6.Latvia | 1 | 0.021 | 0.013897 | 0.002436 | 0.001462 |
| ps5.Latvia:ps8.Latvia | 1 | 0.018154 | 0.006538 | 0.005173 | 0.001808 |
| ps5.Latvia:ps9.Norway | 1 | 0.021726 | 0.00612 | 0.001145 | 0.000598 |
| ps5.Latvia:ps10.Norway | 1 | 0.014188 | 0.00395 | 0.001063 | 0.00045 |
| ps5.Latvia:ps11.Portugal | 1 | 0.010352 | 0.008466 | 0.004114 | 0.004023 |
| ps5.Latvia:ps12.Portugal | 1 | 0.008705 | 0.008474 | 0.007026 | 0.000577 |
| ps5.Latvia:ps14.Portugal | 1 | 0.021165 | 0.005374 | 0.004626 | 0.000626 |
| ps5.Latvia:ps15.Sweden | 1 | 0.006582 | 0.003479 | 0.000788 | 0.000388 |
| ps5.Latvia:ps16.Sweden | 1 | 0.017019 | 0.005971 | 0.003904 | 0.00151 |
| ps6.Latvia:ps7.Latvia | 1 | 0.027918 | 0.011388 | 0.007245 | 0.003367 |
| ps6.Latvia:ps8.Latvia | 1 | 0.031161 | 0.006 | 0.00425 | 0.002643 |
| ps6.Latvia:ps9.Norway | 1 | 0.035476 | 0.009143 | 0.003492 | 0.002683 |
| ps6.Latvia:ps10.Norway | 1 | 0.016341 | 0.013541 | 0.003188 | 0.002835 |
| ps6.Latvia:ps11.Portugal | 1 | 0.018128 | 0.00184 | 0.000947 | 0.000909 |
| ps6.Latvia:ps12.Portugal | 1 | 0.014798 | 0.012679 | 0.002798 | 0.000667 |
| ps6.Latvia:ps13.Portugal | 1 | 0.010802 | 0.010747 | 0.00156 | 0.000275 |
| ps6.Latvia:ps14.Portugal | 1 | 0.016337 | 0.007949 | 0.002031 | 0.001694 |
| ps6.Latvia:ps15.Sweden | 1 | 0.011067 | 0.002267 | 0.001567 | 0.000289 |
| ps6.Latvia:ps16.Sweden | 1 | 0.019563 | 0.006125 | 0.002152 | 0.000696 |
| ps7.Latvia:ps8.Latvia | 1 | 0.031571 | 0.012804 | 0.005054 | 0.001536 |
| ps7.Latvia:ps9.Norway | 1 | 0.020111 | 0.013905 | 0.003746 | 0.001556 |
| ps7.Latvia:ps10.Norway | 1 | 0.013188 | 0.005894 | 0.002718 | 0.002294 |
| ps7.Latvia:ps11.Portugal | 1 | 0.020631 | 0.004733 | 0.000684 | 0.000578 |
| ps7.Latvia:ps12.Portugal | 1 | 0.01969 | 0.009071 | 0.008702 | 0.000143 |
| ps7.Latvia:ps13.Portugal | 1 | 0.018099 | 0.003 | 0.001714 | 0.000725 |
| ps7.Latvia:ps14.Portugal | 1 | 0.021459 | 0.005541 | 0.001429 | 0.00049 |
| ps7.Latvia:ps16.Sweden | 1 | 0.020063 | 0.006518 | 0.001286 | 0.001045 |
| ps8.Latvia:ps9.Norway | 1 | 0.020254 | 0.013492 | 0.002302 | 0.000698 |
| ps8.Latvia:ps11.Portugal | 1 | 0.018834 | 0.006449 | 0.003968 | 0.003059 |
| ps8.Latvia:ps12.Portugal | 1 | 0.023702 | 0.010524 | 0.003131 | 0.000393 |
| ps8.Latvia:ps14.Portugal | 1 | 0.014051 | 0.010561 | 0.004918 | 0.001224 |
| ps8.Latvia:ps15.Sweden | 1 | 0.020678 | 0.003822 | 0.001167 | 0.000156 |
| ps8.Latvia:ps16.Sweden | 1 | 0.017991 | 0.006688 | 0.002036 | 0.001652 |
| ps9.Norway:ps11.Portugal | 1 | 0.024503 | 0.005861 | 0.002075 | 0.000802 |
| ps9.Norway:ps12.Portugal | 1 | 0.023774 | 0.006845 | 0.002048 | 0.000833 |
| ps9.Norway:ps14.Portugal | 1 | 0.013 | 0.010224 | 0.004755 | 0.003327 |
| ps9.Norway:ps15.Sweden | 1 | 0.005178 | 0.0051 | 0.004611 | 0.002267 |
| ps9.Norway:ps16.Sweden | 1 | 0.016321 | 0.008741 | 0.001563 | 0.000973 |
| ps10.Norway:ps12.Portugal | 1 | 0.023422 | 0.006441 | 0.001647 | 0.000922 |
| ps10.Norway:ps14.Portugal | 1 | 0.011176 | 0.008387 | 0.004538 | 0.003697 |

**Table S2 (continued)**

| Pairwise comparison | Rank 1st | Rank 2nd | Rank 3rd | Rank 4th | Rank 5th |
| --- | --- | --- | --- | --- | --- |
| ps10.Norway:ps15.Sweden | 1 | 0.007769 | 0.005582 | 0.001413 | 0.000756 |
| ps10.Norway:ps16.Sweden | 1 | 0.018574 | 0.004882 | 0.003404 | 0.000721 |
| ps11.Portugal:ps12.Portugal | 1 | 0.010657 | 0.003132 | 0.002618 | 0.002196 |
| ps11.Portugal:ps13.Portugal | 1 | 0.004606 | 0.004561 | 0.003484 | 0.001588 |
| ps11.Portugal:ps15.Sweden | 1 | 0.01848 | 0.00088 | 0.000347 | 0.000249 |
| ps11.Portugal:ps16.Sweden | 1 | 0.019338 | 0.00375 | 0.001831 | 0.000684 |
| ps12.Portugal:ps14.Portugal | 1 | 0.010531 | 0.006602 | 0.005388 | 0.001051 |
| ps12.Portugal:ps15.Sweden | 1 | 0.018878 | 0.008622 | 0.002311 | 5.56E-05 |
| ps12.Portugal:ps16.Sweden | 1 | 0.009688 | 0.0055 | 0.005295 | 0.00117 |
| ps13.Portugal:ps14.Portugal | 1 | 0.014663 | 0.012827 | 0.002673 | 0.000449 |
| ps13.Portugal:ps15.Sweden | 1 | 0.003433 | 0.002767 | 0.001156 | 0.001089 |
| ps13.Portugal:ps16.Sweden | 1 | 0.016036 | 0.005786 | 0.002152 | 0.002045 |
| ps14.Portugal:ps15.Sweden | 1 | 0.009922 | 0.007733 | 0.004967 | 0.002544 |
| ps14.Portugal:ps16.Sweden | 1 | 0.017268 | 0.005205 | 0.003304 | 0.000795 |
| ps15.Sweden:ps16.Sweden | 1 | 0.017698 | 0.006573 | 0.002271 | 0.001271 |

**Methods S1** Data preparation - R script

## read data

sppsmlc = read.csv("...\\Desktop\\ps.csv")

## data transformation before imputation

## do box cox transformation on the rest of variables, and get the best lamda for all of them

library(MASS)

nor.sppsmlc.p.raw=data.frame()

for (i in c(2:37)) {

nor.sppsmlc.pi = shapiro.test(sppsmlc[,i])$p.value

nor.sppsmlc.pii = data.frame(variable =names(sppsmlc)[i], p.value =nor.sppsmlc.pi)

nor.sppsmlc.p.raw = rbind(nor.sppsmlc.p.raw,nor.sppsmlc.pii)

}

nor.sppsmlc.p.raw # store the p value of Normal test

n.nor.sppsmlc.p.raw=as.character(nor.sppsmlc.p.raw[nor.sppsmlc.p.raw$p.value>=0.05,]$variable)

n.nor.sppsmlc.p.raw # store variables Normal distributed

non.nor.sppsmlc.p.raw=as.character(nor.sppsmlc.p.raw[nor.sppsmlc.p.raw$p.value<0.05,]$variable)

non.nor.sppsmlc.p.raw # store variables non-Normal distributed

## do box-cox transformation only on Non Normal variables

# get the best lamda

non.nor.sppsmlc.p.raw

non.nor.sppsmlc.p.raw.lamda=data.frame()

for (i in c(1:length(non.nor.sppsmlc.p.raw)) ){

bc.i = boxcox(sppsmlc[non.nor.sppsmlc.p.raw][,i] ~ sppsmlc$variety)

bc.ii = bc.i$x[bc.i$y == max(bc.i$y)]

non.nor.sppsmlc.p.raw.lamdai = data.frame(variable = non.nor.sppsmlc.p.raw[i],lamda =bc.ii)

non.nor.sppsmlc.p.raw.lamda = rbind(non.nor.sppsmlc.p.raw.lamda,non.nor.sppsmlc.p.raw.lamdai )

}

non.nor.sppsmlc.p.raw.lamda # store the best lamda

### box cox transformation

non.nor.sppsmlc.p.bc.tr=sppsmlc[1]

for (i in c(1:length(non.nor.sppsmlc.p.raw)) ){

non.nor.sppsmlc.p.bc.tr00 = sppsmlc[non.nor.sppsmlc.p.raw][,i]^(non.nor.sppsmlc.p.raw.lamda$lamda[i])

non.nor.sppsmlc.p.bc.tr0=data.frame(non.nor.sppsmlc.p.bc.tr00)

non.nor.sppsmlc.p.bc.tr = cbind(non.nor.sppsmlc.p.bc.tr,non.nor.sppsmlc.p.bc.tr0)

}

non.nor.sppsmlc.p.bc.tr # store the box-cox transformed data

names(non.nor.sppsmlc.p.bc.tr)[2:(length(non.nor.sppsmlc.p.raw)+1)]=non.nor.sppsmlc.p.raw # name the newly transformed data

## put Normal variables and box-cox transformed data together

sppsmlct = data.frame(variety=sppsmlc[1],sppsmlc[n.nor.sppsmlc.p.raw],non.nor.sppsmlc.p.bc.tr[-1])

#impute the data with "Amelia" package

# load the "Amelia" and other required packages

library(caret);library(Rcpp);library(Amelia)

library(lattice);library(survival);library(caret)

# multiple imputation 10 times

sppsmlct.out <- amelia(sppsmlct, m = 10, cs="variety")

# get the 10th imputed data

sppsim=sppsmlc2t.out$imputations[[10]]

#oder order variety and give new rownames

sppsimn = sppsim[order(sppsim$variety),]

rownames(sppsimn)= c(1:97)

sppsimn # is used for further analysis

**Methods S2** Flow chart on data processing (RF and SVM combination)


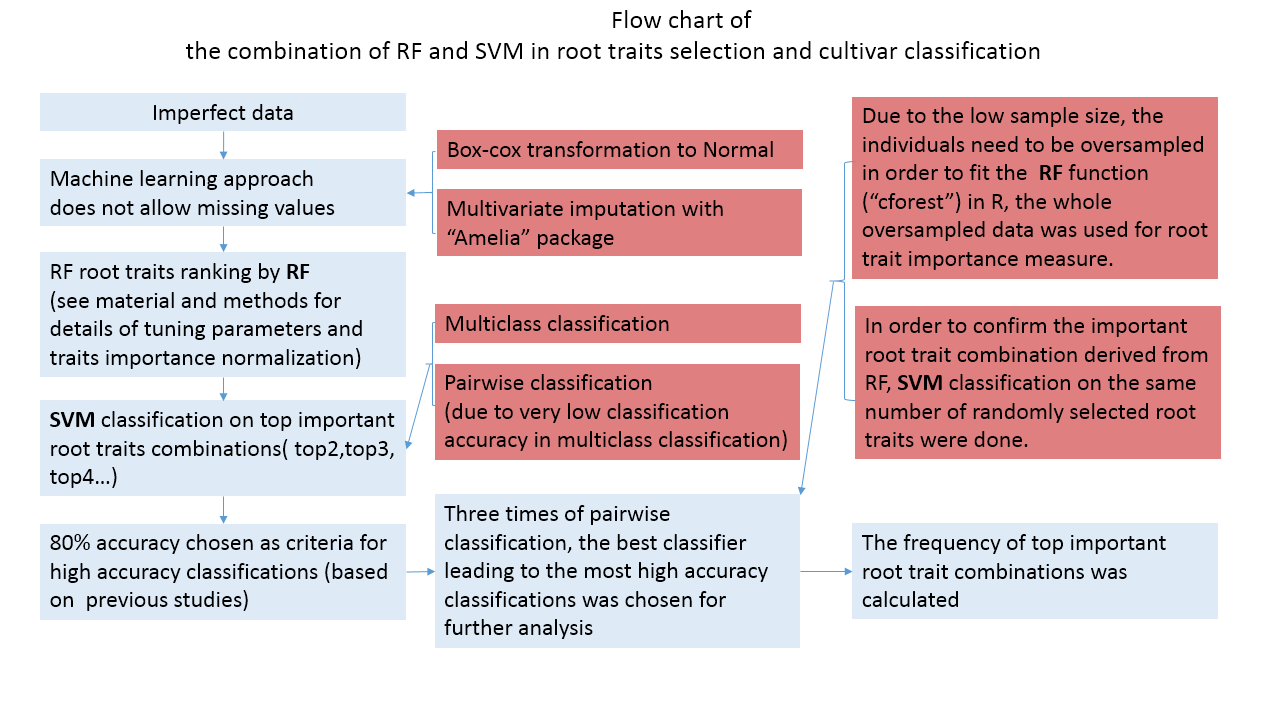


**Methods S3** RF and SVMs analysis - R script

##do random forest variable importance

# 1st multiclass classification

# without root traits selection

tc <- tune.control(cross =nrow(sppsimn))

tune.outm.j <- tune.svm(variety ~., data=sppsimn.s, cost = 10^(-5:6),scale = TRUE,

gamma=10^(-5:6),kernel="radial",tunecontrol = tc,class.weights = wts)

bestcost = tune.outm.j$best.parameters$cost

bestgamma= tune.outm.j$best.parameters$gamma

modelm.j = svm(sppsimn.s$variety ~ ., data = sppsimn,

cost = bestcost,gamma=bestgamma,scale = TRUE,type = "C-classification",

kernel="radial",cross =nrow(sppsimn),probability=TRUE,class.weights = wts)

sm.acc=summary(modelm.j)$tot.acc # save the average accuracy of LOOV

sppsimn.s.multi.or.svm.acc=sm.acc # svm got higher accuracy 18.5%

## RF root traits selection on multiclass problems

sppsimn.multi.im.mtry=data.frame() # variable importance

#summary(sppsimn.multi.or.t)

for (mt in c(1,2,4,6,8,10,12,14,16,18,20,24,28,32)) { # involve different number of mtry s

print(c(mt))

sppsimn.multi.train=sppsimn

sppsimn.multi.train$variety=factor(sppsimn.multi.train$variety)

sppsimn.multi.train.cf <- cforest(variety ~.,

data = sppsimn.multi.train,

control =cforest_unbiased(mtry =mt, ntree = 1000))

sppsimn.multi.im.m.j00=varimp(sppsimn.multi.train.cf, conditional = TRUE) # conditional # permutation test

sppsimn.multi.im.m.j0=data.frame(mtry=mt, t(as.matrix(sppsimn.multi.im.m.j00 )))

sppsimn.multi.im.mtry=rbind(sppsimn.multi.im.mtry,sppsimn.multi.im.m.j0)

}

## SVM on top important root traits combinations

sppsimn.multi.svm.acc=data.frame()

sppsimn.multi.svm.part=data.frame()

for (q in c(1,2,4,6,8,10,12,14,16,18,20,24,28,32)){ # here only tried mtry s give high #accuracy in RF, later may try all mtrys

sppsimn.multi.im.m.j=sppsimn.multi.im.mtry[sppsimn.multi.im.mtry$mtry==q,][-1]

scale.sppsimn.multi.row.mtry.i0=data.frame()

for(p in c(1:nrow(sppsimn.multi.im.m.j))){ # to normalize the variable importance

sppsimn.multi.row.i00= as.numeric(sppsimn.multi.im.m.j[p,])

sppsimn.multi.row.i0= ifelse(sppsimn.multi.row.i00<0,sppsimn.multi.row.i00-abs(min(sppsimn.multi.row.i00)),sppsimn.multi.row.i00)

sppsimn.multi.row.i= ifelse(sppsimn.multi.row.i0<0,0,sppsimn.multi.row.i0)

M.sppsimn.multi.row.i=max(sppsimn.multi.row.i)

SD.sppsimn.multi.row.i=max(sppsimn.multi.row.i)-min(sppsimn.multi.row.i)

scale.sppsimn.multi.row.i00= data.frame(t(as.matrix(sppsimn.multi.row.i/ifelse(SD.sppsimn.multi.row.i==0,1,SD.sppsimn.multi.row.i))))

scale.sppsimn.multi.row.mtry.i0=rbind(scale.sppsimn.multi.row.mtry.i0,scale.sppsimn.multi.row.i00)

}

names(scale.sppsimn.multi.row.mtry.i0)=names(sppsimn.multi.im.mtry)[-(1)]

scale.sppsimn.multi.importance.ob.rank.mtryi =scale.sppsimn.multi.row.mtry.i0

for (imp in c(2,3,5,7,9,11,13,15)) { # to rank the varialbes based on importance

print(c(q,imp))

sppsimn.multi.variable.rank000=scale.sppsimn.multi.importance.ob.rank.mtryi[order(-scale.sppsimn.multi.importance.ob.rank.mtryi)]

sppsimn.multi.variable.rank00=names(sppsimn.multi.variable.rank000)[1:imp] #find top imp important variables

var.rank.m.j= sppsimn.multi.variable.rank00

sppsimn.multi.imn.m.j.or0 = sppsimn[as.character(var.rank.m.j)]

sppsimn.multi.imn.m.j.or=data.frame(variety=sppsimn[1],sppsimn.multi.imn.m.j.or0)

tc <- tune.control(cross =nrow(sppsimn.multi.imn.m.j.or))

tune.outm.j <- tune.svm(variety ~., data=sppsimn.multi.imn.m.j.or, cost = 10^(-5:6),

gamma=10^(-5:6),kernel="radial",tunecontrol = tc)

bestcost = tune.outm.j$best.parameters$cost

bestgamma= tune.outm.j$best.parameters$gamma

modelm.j = svm(sppsimn.multi.imn.m.j.or$variety ~ ., data = sppsimn.multi.imn.m.j.or,

cost = bestcost,gamma=bestgamma,scale = TRUE,type = "C-classification",

kernel="radial",cross =nrow(sppsimn.multi.imn.m.j.or),probability=TRUE)

sm.acc.0=summary(modelm.j)$tot.acc # save the average accuracy of LOOV

sm.part.0=summary(modelm.j)$accuracies

sm.acc=data.frame(q=q,imp=imp,sm.acc.0)

sm.part=data.frame(t(sm.part.0))

sppsimn.multi.svm.acc=rbind(sppsimn.multi.svm.acc,sm.acc)

sppsimn.multi.svm.part=rbind(sppsimn.multi.svm.part,sm.part)

}

}

# 2nd pairwise classification

library(party) # package for random forest

###

spps.rfac.m.j.mtry1_3 = data.frame() # to store accuracy of random forest

for (r in c(1:3)){ # root trait ranking for 3 times

for (mt in c(1,2,4,6,8,10,12,14,16,18,20,24,28,32)) { # involve different number of mtry s

for (m in c(1:15)) {

for (j in c((m+1):16)) {

print(c(r,mt,m,j)) # get the idea of data processing

sppsimn.m.j=sppsimn[sppsimn$variety%in%c(m,j),]

# divide data into training and testing

sppsimn.m.j.train=rbind(sppsimn.m.j,sppsimn.m.j,sppsimn.m.j,sppsimn.m.j)

sppsimn.m.j.train$variety=factor(sppsimn.m.j.train$variety)

# do random forest analysis

sppsimn.m.j.train.cf <- cforest(variety ~ .,

data = sppsimn.m.j.train,

control =cforest_unbiased(mtry =mt, ntree = 1000))

spps.im.m.j00=varimp(sppsimn.m.j.train.cf, conditional = TRUE) # conditional permutation importance

# store variable importance

spps.im.m.j0=data.frame(run=r,mtry=mt, round=m, var=j, t(as.matrix(spps.im.m.j00 )))

spps.im.m.j.mtry1_3=rbind(spps.im.m.j.mtry1_3,spps.im.m.j0)

}

}

}

}

# load required packages

library(ggplot2)；library(reshape2)；library(e1071)；library(lattice)；library(MASS)

M.ps.acm.j.or.mtryi.impi.1_3=data.frame() # store the accuracy of SVMs

for ( ru in c(1:3)){ # classification done three times based on ranked root traits

spps.im.m.j.mtry=spps.im.m.j.mtry1_3[spps.im.m.j.mtry1_3$run==ru,]

for (q in c(1,2,4,6,8,10,12,14,16,18,20,24,28,32)){ # different numbers of mtrys

spps.im.m.j=spps.im.m.j.mtry[spps.im.m.j.mtry$mtry==q,][-c(1,2)]

scale.spps.row.mtry.i0=data.frame()

for(p in c(1:nrow(spps.im.m.j))){ # to normalize the variable importance

spps.row.i00= as.numeric(spps.im.m.j[p,-c(1,2)])

spps.row.i0= ifelse(spps.row.i00<0,spps.row.i00-abs(min(spps.row.i00)),spps.row.i00)

spps.row.i= ifelse(spps.row.i0<0,0,spps.row.i0)

SD.spps.row.i=max(spps.row.i)-min(spps.row.i)

scale.spps.row.i00= data.frame(t(as.matrix(spps.row.i/ifelse(SD.spps.row.i==0,1,SD.spps.row.i))))

scale.spps.row.mtry.i0=rbind(scale.spps.row.mtry.i0,scale.spps.row.i00)

}

names(scale.spps.row.mtry.i0)=names(spps.im.m.j.mtry)[-(1:4)]

scale.spps.importance.ob.rank.mtryi= scale.spps.row.mtry.i0 # just pass names

for (imp in c(2,3,5,7,9,11,13,15)) { # to different combination of top important variables

spps.variable.rank1=data.frame()

for (i in c(1:120)) {

spps.variable.rank000=scale.spps.importance.ob.rank.mtryi[i,][order(-scale.spps.importance.ob.rank.mtryi[i,])]

spps.variable.rank00=names(spps.variable.rank000)[1:imp] #find top imp important variables

spps.variable.rank0=data.frame(rown=i,transpose(as.matrix(spps.variable.rank00 )))

spps.variable.rank1=rbind(spps.variable.rank1,spps.variable.rank0)

}

spps.variable.rank=cbind(spps.im.m.j.mtry[1:120,3:4],spps.variable.rank1[-1])

for (m in c(1:15)) { # pass important variables to rbf SVMs

for (j in c((m+1):16)) {

print(c(ru,q,imp,m,j)) # get the idea of data processing

##################

var.rank.m.j=spps.variable.rank[spps.variable.rank$round==m,][spps.variable.rank[spps.variable.rank$round==m,]$var==j,]

sppsimn.m.j.or0 = sppsimn[sppsimn$variety %in% c(m,j),][as.character(unname(unlist(var.rank.m.j[-c(1:2)])))]

sppsimn.m.j.or=data.frame(variety=factor(sppsimn[sppsimn$variety %in% c(m,j),]$variety),

sppsimn.m.j.or0)

tc <- tune.control(cross =nrow(sppsimn.m.j.or))

# tune the best cost and gamma parameters

tune.outm.j <- tune.svm(variety ~., data=sppsimn.m.j.or, cost = 10^(-5:6),

gamma=10^(-5:6),kernel="radial",tunecontrol = tc)

bestcost = tune.outm.j$best.parameters$cost

bestgamma= tune.outm.j$best.parameters$gamma

# run rbf SVMs with LOOCV

modelm.j = svm(sppsimn.m.j.or$variety ~ ., data = sppsimn.m.j.or[,-1],

cost = bestcost,gamma=bestgamma,scale = TRUE,type = "C-classification",

kernel="radial",cross =nrow(sppsimn.m.j.or),probability=TRUE)

no.row = nrow(sppsimn.m.j.or)

sm.acc=summary(modelm.j)$tot.acc # store the overal accuracy of LOOV

M.ps.acm.j.or0=data.frame(runi=ru, mtry=q,impi=imp,round=m, var=j, Mo.acc=sm.acc)

M.ps.acm.j.or.mtryi.impi.1_3=rbind(M.ps.acm.j.or.mtryi.impi.1_3,M.ps.acm.j.or0)

}

}

}

}

}

####################

## prediction accuracy stored

M.ps.acm.j.or.mtryi.impi.1_3

**Methods S4** Univariate permutation test – R script

#### univariate analysis

# load data

sppsimn=read.csv("sppsimn.csv")

library(EnvStats) # load the library for permutation test

# permutation to get the null distribution of difference of means

names(sppsimn)

p.value.0=data.frame()

for (m in c(1:15)) {

for (j in c((m+1):16)) {

print(c(m,j))

##################

sppsimn.m.j.or.m = sppsimn[sppsimn$variety ==m,]

sppsimn.m.j.or.j = sppsimn[sppsimn$variety==j,]

# here I do exact permutation, first get the number of possible combinations

sppsimn.m.j.or.m$variety=factor(sppsimn.m.j.or.m$variety)

sppsimn.m.j.or.j$variety=factor(sppsimn.m.j.or.j$variety)

p.value.00=c()

for (k in c(2:37)){

p.value.000=twoSamplePermutationTestLocation(sppsimn.m.j.or.m[,k],

sppsimn.m.j.or.j[,k],

fcn = "mean", alternative = "two.sided",

mu1.minus.mu2 = 0, paired = FALSE, exact = T,

# n.permutations = 1000,

seed =123, tol = sqrt(.Machine$double.eps))$p.value

p.value.00=c(p.value.00,p.value.000)

}

p.value.0=rbind(p.value.0,p.value.00)

}

}

####

ppsw.t=p.value.0

# give new row and column names

names(ppsw.t)=names(sppsimn)[2:37]

ppsw.t.f=data.frame(ps.group,ppsw.t)

#rownames(ppsw.t.f)=c(1:120)

ppsw.t.f # p values of all comparisons

## bonferroni correction

ppsw.t.f.bof0=data.frame()

for (i in c(1: nrow(ppsw.t.f))){

ppsw.t.f.bof00=p.adjust(ppsw.t.f[i,-1], method = "bonferroni", n = ncol(ppsw.t.f[,-1]))

ppsw.t.f.bof0=rbind(ppsw.t.f.bof0,ppsw.t.f.bof00)

}

names(ppsw.t.f.bof0)=names(ppsw.t.f)[-1]

ppsw.t.f.bof=data.frame(ppsw.t.f[1],ppsw.t.f.bof0 )

## "fdr" correction

ppsw.t.f.fdr0=data.frame()

for (i in c(1: nrow(ppsw.t.f))){

ppsw.t.f.fdr00=p.adjust(ppsw.t.f[i,-1], method = "fdr", n = ncol(ppsw.t.f[,-1]))

ppsw.t.f.fdr0=rbind(ppsw.t.f.fdr0,ppsw.t.f.fdr00)

}

names(ppsw.t.f.fdr0)=names(ppsw.t.f)[-1]

ppsw.t.f.fdr=data.frame(ppsw.t.f[1],ppsw.t.f.fdr0 )

##

# put accuracy + univariate p value together

M.ps.acm.j.or.mtry24.imp5 # data frame containing SVM prediction accuracy

uni.spps.acc=data.frame(M.ps.acm.j.or.mtry24.imp5[5])# extract accuracy only

## bof correction

ppsw.t.f.bof # bonferroni corrected p values

uni.spps.acc.t.bof0 =data.frame(ppsw.t.f.bof[1],uni.spps.acc,ppsw.t.f.bof[-1])

uni.spps.acc.t.bof=uni.spps.acc.t.bof0 # pass names

## fdr correction

ppsw.t.f.fdr # fdr corrected p values

uni.spps.acc.t.fdr0 =data.frame(ppsw.t.f.fdr[1],uni.spps.acc,ppsw.t.f.fdr[-1])

uni.spps.acc.t.fdr=uni.spps.acc.t.fdr0 # pass names
